# Molecular Mining of Alleles in Water Buffalo *Bubalus bubalis* and Characterization of the *TSPY1* and *COL6A1* Genes

**DOI:** 10.1371/journal.pone.0024958

**Published:** 2011-09-15

**Authors:** Sudeep Kumar, Ruchi Gupta, Sudhir Kumar, Sher Ali

**Affiliations:** 1 Molecular Genetics Laboratory, National Institute of Immunology, New Delhi, Delhi, India; 2 Department of Zoology, University of Lucknow, Lucknow, Uttar Pradesh, India; The University of Maryland, United States of America

## Abstract

**Background:**

Minisatellites are an integral part of eukaryotic genomes and show variation in the complexity of their organization. Besides their presence in non-coding regions, a small fraction of them are part of the transcriptome, possibly participating in gene regulation, expression and silencing. We studied the minisatellite (TGG)_n_ tagged transcriptome in the water buffalo *Bubalus bubalis* across various tissues and the spermatozoa, and characterized the genes *TSPY1* and *COL6A1* discovered in the process.

**Results:**

Minisatellite associated sequence amplification (MASA) conducted using cDNA and oligonucleotide primer (TGG)_5_ uncovered 38 different mRNA transcripts from somatic tissues and gonads and 15 from spermatozoa. These mRNA transcripts corresponded to several known and novel genes. The majority of the transcripts showed the highest level of expression either in the testes or spermatozoa with exception of a few showing higher expression levels in the lungs and liver. Transcript SR1, which is expressed in all the somatic tissues and gonads, was found to be similar to the *Bos taurus* collagen type VI alpha 1 gene (*COL6A1*). Similarly, SR29, a testis-specific transcript, was found to be similar to the *Bos taurus* testis-specific Y-encoded protein-1 representing cancer/testis antigen 78 (CT78). Subsequently, full length coding sequences (cds) of these two transcripts were obtained. Quantitative PCR (q-PCR) revealed 182-202 copies of the*TSPY1* gene in water buffalo, which localized to the Y chromosome.

**Conclusions:**

The MASA approach enabled us to identify several genes, including two of clinical significance, without screening an entire cDNA library. Genes identified with TGG repeats are not part of a specific family of proteins and instead are distributed randomly throughout the genome. Genes showing elevated expression in the testes and spermatozoa may prove to be potential candidates for in-depth characterization. Furthermore, their possible involvement in fertility or lack thereof would augment animal biotechnology.

## Introduction

Satellite DNA, an integral part of eukaryotic genomes [1], present as long uninterrupted arrays, often in genetically silent heterochromatic regions [2]. These dynamic elements include transposable elements, major satellites and simple sequence repeats (SSRs) [2], [3] and represent a fast-evolving part of the genome conforming to the random processes of molecular drive [4]. Satellite sequences are involved in both gene conversion and unequal crossing over. These events are responsible for the rapid horizontal spread of mutations [5], changes in copy number and even the loss of satellite sequences from the genome. Owing to these rearrangements, copy number variation is caused even amongst the closely related species [6], [7].

Usually, satellites are present in non-coding regions but a small fraction can be found in the transcriptome [8], [9] and this subset participates in gene regulation and silencing [10], [11]. In the context of disease, pathogenic trimeric repeat expansion in humans has been well established. Similar structures may act as substrates for genome-wide pathogenic rearrangements [12]. The expansion and contraction of these SSRs within the exonic regions are reported to cause several diseases, such as Myotonic dystrophy, Huntington's disease and fragile X syndrome [13]–[15]. Further, the presence of ITRs (internal tandem repeats) at exon-intron boundaries may give rise to novel alternatively spliced transcripts [16]. Notwithstanding these observations, the precise arrangement of tandem repeats in a given species in the context of genomic organization and gene expression still remains unclear.

Another aspect of gene expression relates to germline genetics. In the past, the spermatozoon was considered to be merely the carrier of the paternal genome. However, this perception has changed since it was discovered that spermatozoa contribute (except in mice) a centriole [17] and a soluble factor that activates the egg [18]. Despite being in a transcriptionally dormant state [19], spermatozoa retain a pool of mRNAs. These messages are transcribed long before nuclear shutdown [20]–[22] and encode the proteins needed for the subsequent re-packaging of DNA and micro-RNAs [23]. Approximately 3,000–5,000 mRNA transcripts have been reported to be present in spermatozoa [21], [23]–[27]. As spermatozoon development results in the loss of rRNA, translation in spermatozoa is not possible. The delivery of the spermatozoal transcripts to the ooplasm is hypothesized to have biological significance during fertilization, embryogenesis and subsequent morphogenesis. However, the spermatozoon's genomic organization, cellular expression and association with regulatory elements remain unexplored.

In exons, trinucleotide repeats are favored evolutionarily due to selection against frame shift mutations [28]. These repeats could serve as markers to discover novel genes [29]. The tandem repeat length polymorphism of (CCA)n/(TGG)n resulting in conformational variability of the DNA sequence is well documented in the human genome [30]. We have used (TGG)_5_ repeats to uncover somatic, gonadal and spermatozoal transcripts in the water buffalo *Bubalus bubalis*, which is an important livestock animal in the Indian subcontinent. Thus far, this repeat has been studied in the context of human genetic diseases but it has not been studied in a non-human system. The unexplored status of the water buffalo genome makes molecular mining of the alleles even more relevant. The detailed insight into the repeat tagged mRNA transcripts across the tissues including spermatozoa in the water buffalo appears to be the first such study. This expression profile is expected to increase our understanding of the involvement of minisatellites in the regulation of gene expression in a tissue specific manner.

## Materials and Methods

### Ethics statement

Tissue samples from both sexes of water buffalo were collected from the Gazipur slaughter house, New Delhi, India, with the help of an on-site veterinary officer. Fresh water buffalo semen samples were procured from an *in-vitro* fertilization (IVF) center (Frozen Semen Production Center, Chak Gajaria), in Lucknow (U.P), India. These collections were performed in accordance with the guidelines of the Institute's Ethical and Bio-safety committees. There was no additional requirement for use of these samples. Therefore, any additional approvals were not applicable in this case.

### Sperm purification and RNA isolation

Fresh water buffalo semen samples were procured from the IVF center as described above and utmost care was taken to avoid diploid cell contamination. Collected samples were subjected to the percoll gradient method to select for motile sperm. RNA isolation was performed using TRIzol reagent (Sigma-Aldrich) following standard protocols [20], [31], [32]. Isolated RNA was quantified using a spectrophotometer (Amersham Life Sciences) and tested for the presence of residual DNA by PCR using primers against beta-actin (*ACTB*; [GenBank: DQ661647]) and Protamine-1 (*PRM1*; [GenBank: NM_174156]) following standard procedures [20], [32] (Table S1). Subsequently, cDNA synthesis was performed using a high capacity cDNA reverse transcription kit (ABI, USA). The absence of non-sperm cells in the processed semen samples was confirmed by PCR using primers specific to common leukocyte antigen (*CD45*) and epithelial E-cadherin (*CDH1*) gene markers (Table S1) as described previously [33].

### Total RNA isolation and cDNA synthesis from tissue samples

Tissue samples from both sexes of water buffalo were collected from the local slaughter house as described above. Total RNA was isolated from cardiac, renal, hepatic, pulmonary, splenic, testicular and ovarian tissue using TRIzol reagent (Sigma-Aldrich) following standard protocols [20], [31], [32]. After quantification of the RNA by spectrophotometry, each sample was tested for genomic DNA contamination by PCR using primers specific to beta-actin (*ACTB*) [GenBank: DQ661647]. Synthesis of cDNA was conducted using a high capacity cDNA reverse transcription kit (ABI, USA). The quality of the cDNA produced was confirmed by PCR amplification using beta-actin primers.

### Minisatellite associated sequence amplification (MASA)

**Figure 1:**
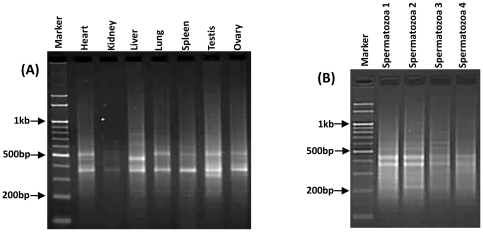
Minisatellite associated sequence amplification conducted using decoy primer (TGG)_5_. Panel (**A**)shows bands uncovered by MASA from different somatic tissues and gonads and (**B**) from spermatozoa. The corresponding tissues are indicated above.

To conduct MASA, a 15 bp oligo (5' TGGTGGTGGTGGTGG 3') was purchased from Sigma-Aldrich. MASA reactions were performed using cDNA templates from different somatic and gonadal tissues of two individuals and from spermatozoa of four individuals following standard procedures [32], [34]. Representative gel pictures are shown in figure 1. The annealing temperature of the repeat primer was 52°C. The resultant amplicons were resolved on a 2% (w/v) agarose gel using 1x TAE buffer.

### Cloning, sequencing and characterization of MASA amplicons

After the MASA reactions, the amplicons resolved on the agarose gel were excised. DNA was eluted (Qiagen Gel Extraction kit, Germany), cloned into the pGEMT-easy vector (Promega, USA) and used to transform DH5-alpha cells. The positive clones were identified by *Eco*R1 digestion (New England Biolabs). All of the sequencing reactions were performed on the Applied Biosystems 3130*xl* Genetic Analyzer. It used an initial cycle sequencing reaction mixture of 10 µl with BigDye®Terminator V3.1 cycle sequencing RR-100. Subsequent product was purified by ethanol/EDTA/sodium acetate precipitation as per the manufacturer's instructions. Finally, the precipitate was resuspended in 10 µl of Hi-Di™ Formamide Genetic Analysis Grade. Gel electrophoresis was run on a 36 cm capillary array with POP-7TM polymer. The raw data obtained was analyzed with the Genetic Analyzer Data collection Software v3.0. Multiple clones were sequenced to validate the obtained sequences, which were then deposited in GenBank. A database search was conducted to determine the homology of these sequences with other entries in GenBank using the default server [35] with the megablast “highly similar” and blastn “somewhat similar” algorithms. Each sequence was first subjected to blast search across the database of reference mRNA sequences (refseq_mRNA), then against the nucleotide collection (nr/nt) of all organisms, and finally to those of *Bos taurus* (Table S2).

### RT-PCR and relative expressional studies using q-PCR

**Figure 2:**
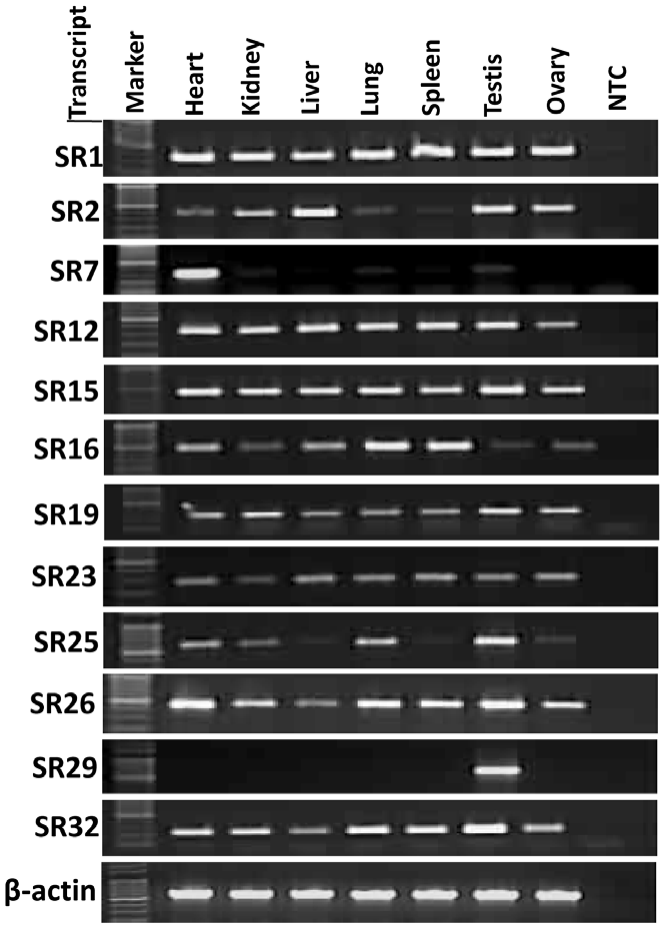
RT-PCR analysis of the (TGG)_n_ tagged mRNA transcripts using internal primers and cDNA from different somatic tissues and the gonads. The quality and quantity of the cDNA samples were assessed using beta-actin specific primers and are shown at the bottom. The transcript IDs are indicated on the left and the tissues IDs at the top of the panel. NTC denotes non-template control.

The expression status of the identified genes was determined in the different tissues and spermatozoa by both RT-PCR and q-PCR. For this, equal amounts of RNA from the different tissues were reverse transcribed into cDNA. An expression profile was ascertained by RT-PCR (Figure 2) using this cDNA and internal primers (Table S3). These primers were designed from the individual mRNA transcripts using Primer 3 Input (version 0.4.0). Beta-actin was used as an endogenous control.

**Figure 3:**
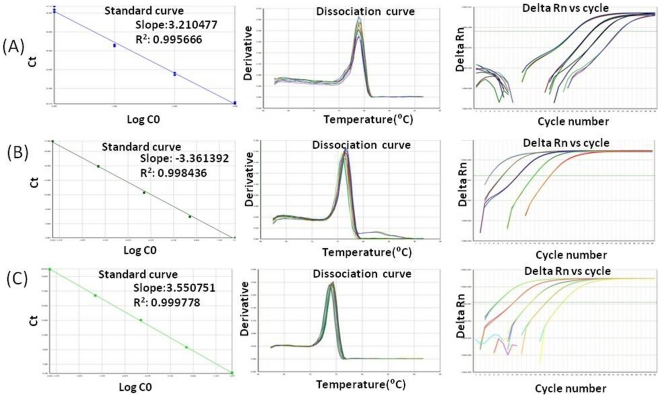
Representative standard and dissociation curves and amplification plots from Real Time PCR assays. Melting (dissociation) curve showing a single peak corresponds to a single amplicon. (**A**) Representative curves for *GAPDH*, (**B**) RS3 and (**C**) RS5.

To compare the relative expression of different genes and gene fragments, SYBR green assays were conducted using Real Time PCR (Sequence Detection System, 7500, ABI) according to established protocols [34], [36]. *GAPDH* (Glyceraldehyde 3-phosphate dehydrogenase) was used as an endogenous control. Primers for determining relative expression (Table S4) for each of the transcripts were designed with “Primer Express Software” (ABI, USA). The q-PCR assay was performed individually for all transcripts by Real Time PCR using Power SYBR® green (Part No. 4367659, ABI). For each transcript, a calibrator tissue that showed basal expression level 1 was chosen. This calibrator was selected based on the lowest expression of that transcript in the tissues studied. Primer specificity and comparable PCR efficiencies for all of the studied genes and the endogenous control (*GAPDH*) were ensured. For this, standard and melting curves were generated using ten-fold serial dilutions of the cDNA templates. Standard curves with a slope ranging from 3.3 to −3.6, R^2^>0.99 (Regression coefficient) were considered to have acceptable PCR efficiencies, and a single dissociation peak indicated primer specificity (Figure 3). The expression level of the genes was calculated using this formula: relative expression  =  (1+E)^−^^ΔΔCt^, where E is the efficiency of the PCR and ΔΔCt is the cycle threshold normalized first with the endogenous control *GAPDH* (Ct sample – Ct *GAPDH*  =  ΔCt) and then with the calibrator sample (ΔCt Sample - ΔCt Calibrator  =  ΔΔCt).

### Amplification of the full length coding sequence of identified transcripts

The amplicon SR1 [GenBank: GU433053] of *Bubalus bubalis* is a partial cds showing >95% identity with *Bos taurus* collagen type VI alpha 1 (*COL6A1*) mRNA. The full length SR1 transcript was amplified using cDNA from a *Bubalus bubalis* ovary with primers (Table S5) designed from the *Bos taurusCOL6A1* coding sequence [GenBank: NM_001143865.1]. The PCR amplicons were cloned and subsequent sequencing detected four overlapping clones. These sequences were aligned to obtain the full length cds of *COL6A1* of *Bubalus bubalis*.

The amplicon SR29 [GenBank: GU433091], a partial cds, showed >95% identity with *Bos taurus* testis-specific Y-encoded protein-1 representing cancer/testis antigen 78 (CT78) mRNA [GenBank: XM_001254382.2]. The full length sequence of this gene was obtained using the 3′ RACE System for Rapid Amplification of cDNA ends (Invitrogen) and with a gene specific primer (Table S5) designed for the 5′ start site of *Bos taurus TSPY1*-like mRNA. The final product was ligated into the pJET1.2/blunt cloning vector (Fermentas) and was used to transform DH5- alpha *E. coli* cells. Sequencing of this fragment was done commercially (Bioserve Biotechnologies Pvt. Ltd., India).

### Chromosomal localization of the*TSPY1*-like gene by Fluorescence *in situ* hybridization (FISH)

Water buffalo metaphase chromosomes were prepared according to the standard protocol [37]. FISH was conducted using *Bos taurus* Y Chr CH240-127C20 BAC (bacterial artificial chromosome) [GenBank: AC234853.4] procured from BACPAC (Resource Centre, Oakland, California, USA). The BAC DNA was verified with gene specific primers by end point PCR. The BAC DNA was labeled with Fluorescein tagged dUTP (Invitrogen) using the Nick Translation Kit from Abott Molecular Inc. (IL, USA). FISH was subsequently conducted following established protocols [38]. The slides were screened under an Olympus fluorescence microscope (BX 51) fitted with a vertical fluorescence illuminator U-LH100HG UV, excitation and barrier filters. The images were captured and analyzed with Applied Imaging Systems Cytovision 3.92. Chromosomal identification was performed in accordance with ISCNDB 2000 (International system for chromosome nomenclature of domestic bovids) [39].

### Copy number calculation of the*TSPY1*-like gene in the water buffalo genome

The copy number of the *Bubalus bubalis TSPY1*-like gene was calculated using a SYBR green assay with the Real Time PCR Sequence detection system 7500 (ABI, USA) as per the standard protocol [40]. Briefly, a standard curve was generated using 10-fold serial dilutions of the recombinant plasmids in the range of 3,000 to 3,00 million copies. The copy number of the gene in the water buffalo genome was estimated by extrapolation from the standard curve.

## Results

### (TGG)_n_ tagged mRNA transcripts include several known and novel genes

MASA conducted with cDNA from somatic and gonadal tissues of two individuals using a (TGG)_5_ repeat primer uncovered 41 amplicons, and the same primer yielded 28 amplicons from the spermatozoa derived from 4 different animals. Cloning, sequencing and subsequent bioinformatics analysis of these amplicons resulted in the identification of 38 distinct mRNA transcripts corresponding to different genes from the somatic tissues and gonads and an additional 15 from the spermatozoa. Based on RT-PCR, SR1 was found to have similar expression levels in all the somatic and gonadal tissues studied, while the other transcripts showed varying expression across these tissues. Of the 15 spermatozoal transcripts, only one was detected in all the somatic tissues. Thus, the expression of 14 mRNA transcripts was exclusive to spermatozoa.

Of the 38 transcripts from somatic and gonadal tissues, 18 showed significant identity with cDNA sequences from the refseq_mRNA database. Of these, 14 had 90–99% query coverage (Table S6). The remaining ones had regions lacking identity at either the 5′/3′ region or the intervening sequences of the characterized genes. Surprisingly, none of the spermatozoa-specific mRNA transcripts had significant identity with any of the sequences in the database, suggesting that these genes have yet to be characterized.

### Differential expression of the (TGG)_n_ tagged mRNA transcripts

**Figure 4:**
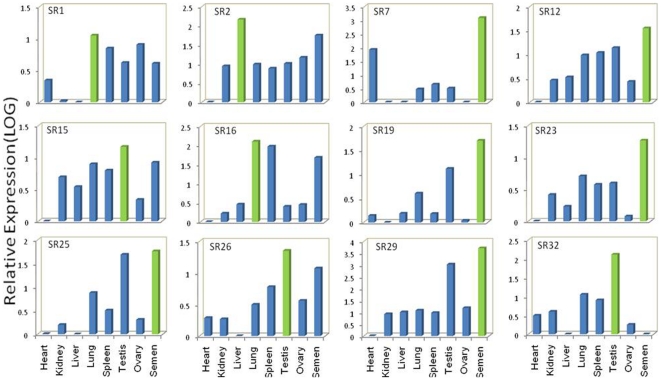
Quantification of mRNA transcripts originating from different tissues. The bar represents the expression level of the transcripts, which are labeled at the top left. The tissue IDs are displayed at the bottom. Maximum expression observed in a tissue is shown in green. To obtain a comparative profile, subsequent q-PCR using internal primers was conducted on all cDNA samples including that from semen. Note the highest expression level of most of the fragments occurs in the testes or spermatozoa.

Significantly, of the 38 mRNA transcripts from somatic and gonadal tissues, about ∼90% showed the highest level of expression in the testes and spermatozoa, while an additional 8% were most highly expressed in the lungs. One (SR2) [GenBank: GU433054] showed the highest level of expression in liver. Of all the transcripts first identified in the testes, only one (SR29) [GenBank: GU433091] showed the highest level of expression in the spermatozoa (5113.16-fold higher as compared with samples from the heart, which was used as the calibrator representing basal level 1), although the testes did show the second highest level of expression (1052.8-fold) for this transcript. The transcript was similar to that of the *Bos taurus* Testis-specific Y-encoded protein 1 (Cancer/testis antigen 78) (CT78). Expression of the transcript SR7 [GenBank: GU433064] was confined mainly to the heart and spermatozoa. This transcript showing the highest level of expression in the heart was found to be homologous with the *Equus caballus* gene for beta-myosin heavy chain [GenBank: D84227.1]. Details from the expression analysis of the transcripts detected in tissue samples, their corresponding accession numbers and relative expression (expressed in folds) are given in table 1. Graphical representation of the expression of a few of the transcripts is shown in figure 4.

**Table 1:** Relative expression for all the TGG tagged transcripts uncovered from somatic tissues and gonads of water buffalo *Bubalus bubalis.*

Transcript ID	Clone ID	Accession nos	Heart	Kidney	Liver	Lung	Spleen	Testis	Ovary	Semen
SR1	pSRC1	GU433047	2.20	1.05	Cb	11.16	7.01	4.17	8	4.06
SR2	pSRC8	GU433054	Cb	8.75	145.01	9.78	7.62	10.20	14.72	56.10
SR3	pSRC13	GU433059	2.69	4.47	2.95	25.46	8	2.19	Cb	572.05
SR4	pSRC15	GU433061	1.87	2.39	3.71	Cb	1.34	1.61	-	396.18
SR5	pSRC16	GU433062	33.13	3.71	2.20	6.15	10.06	4.69	Cb	190.02
SR6	pSRC17	GU433063	5.90	9.45	5.94	46.21	39.67	34.30	6.63	Cb
SR7	pSRC18	GU433064	83.87	Cb	-	2.99	4.47	3.23	-	1226.2
SR8	pSRC19	GU433065	1.07	2	1.88	6.96	6.96	2.97	Cb	580.04
SR9	pSRC20	GU433066	3.95	7.94	11.24	29.45	32.45	3.27	Cb	3213.6
SR10	pSRC22	GU433068	Cb	2.20	1.72	6.96	5.78	13.0	1.57	-
SR11	pSRC23	GU433069	Cb	2.03	2.11	8.88	11.47	11.88	2.81	70.03
SR12	pSRC24	GU433070	Cb	2.87	3.34	9.65	10.85	13.74	2.69	35.26
SR13	pSRC26	GU433072	Cb	2.50	1.47	6.41	2.43	1.24	1.06	37.27
SR14	pSRC27	GU433073	Cb	1.15	21.41	6.73	1.56	1.59	1.96	474.41
SR15	pSRC28	GU433074	Cb	4.92	3.46	7.89	6.28	14.72	2.17	8.34
SR16	pSRC30	GU433076	Cb	1.66	2.87	126.24	93.70	2.51	2.81	47.84
SR17	pSRC31	GU433077	Cb	1.15	2.60	9.92	11.63	5.13	-	572.05
SR18	pSRC32	GU433078	3.16	7.41	Cb	13.09	12.38	188.71	1.97	2105.58
SR19	pSRC34	GU433080	1.39	Cb	1.54	4.03	1.52	13.18	1.09	50.56
SR20	pSRC36	GU433082	1.82	3.76	Cb	18.77	23.92	1.78	1.79	24.42
SR21	pSRC37	GU433083	Cb	2.68	2.43	12.47	9.65	20.39	1.31	-
SR22	pSRC38	GU433084	Cb	2.81	15.14	12.21	12.21	51.27	11.71	471.14
SR23	pSRC39	GU433085	Cb	2.60	1.71	5.10	3.76	3.94	1.20	18.51
SR24	pSRC40	GU433086	2.69	2.79	Cb	7.89	30.27	7.62	10.20	537.45
SR25	pSRC41	GU433087	1.01	1.58	Cb	7.57	3.20	48.84	2.03	57.68
SR26	pSRC42	GU433088	1.93	1.85	Cb	3.14	5.98	22.47	3.63	11.79
SR27	pSRC43	GU433089	Cb	2.89	3.25	6.41	5.62	1.40	1	69.55
SR28	pSRC44	GU433090	1.87	1.64	1.34	5.94	2.91	3.56	Cb	174.85
SR29	pSRC45	GU433091	Cb	8.40	10.13	11.96	9.51	1052.8	15.24	5113.16
SR30	pSRC46	GU433092	Cb	2	3.58	7.06	5.82	4	1.71	137.19
SR31	pSRC47	GU433093	Cb	5.90	27.67	5.82	18.51	12.13	3.73	2538.92
SR32	pSRC48	GU433094	3.12	3.97	Cb	11.24	8	129.79	1.79	-
SR33	pSRC49	GU433095	1.25	2.60	1.89	4.47	6.45	2.19	Cb	1038.30
SR34	pSRC50	GU433096	1.85	Cb	2.44	10.37	7.29	6.60	8.18	54.66
SR35	pSRC51	GU433097	Cb	1.06	2.39	67.18	70.52	9.45	5.50	916.50
SR36	pSRC53	GU433099	1.18	2.34	1.81	7	4.23	3.43	Cb	621.43
SR37	pSRC54	GU433100	Cb	6.50	1.74	15.56	27.47	146.01	12.55	-
SR38	pSRC55	GU433101	1.06	3.61	-	2.55	2.97	29.86	Cb	147.03

In this table, Cb represents the calibrator tissue (expression value 1) showing least expression with which comparisons for the expression in other tissues was made. The value in each row signifies the corresponding fold of expression which is higher as compared to the calibrator value.

**Figure 5:**
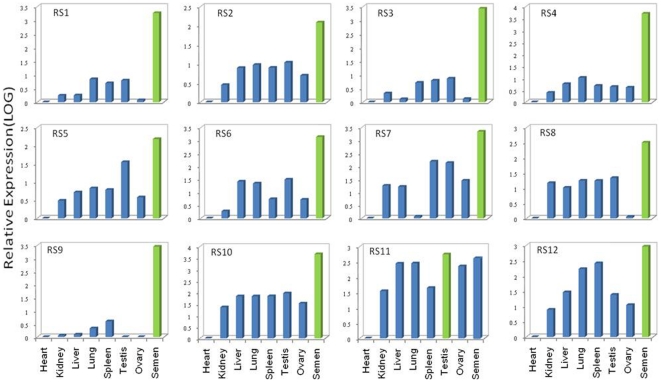
Quantification of mRNA transcripts originating from semen samples. Except forRS11, all mRNA transcripts were most highly expressed in the semen samples (green). To obtain a comparative profile, subsequent q-PCR using internal primers was conducted on all cDNA samples.

Of the 15 transcripts identified in the spermatozoa, expression studies (Table 2) were performed on only 12 because one had already been identified in the tissues sampled and the other two did not have significant ct values (≥40) to be considered for further study. RS11 was most highly expressed in the testes, whereas the remaining 11 showed the greatest level of expression in the spermatozoa. Details from the expression analysis of these 12 spermatozoal mRNA transcripts are shown in figure 5. Notably, 80% of these transcripts were found to have negligible expression in the ovaries, which supports our hypothesis that these transcripts have male-specific functions.

**Table 2:** Relative quantitative expression of TGG tagged transcripts uncovered from the spermatozoa of Buffalo *Bubalus bubalis.*

Transcript ID	Clone ID	Accession nos	Heart	Kidney	Liver	Lung	Spleen	Testis	Ovary	Semen
RS1	pRSC1	GU391953	Cb	1.75	1.79	6.96	4.96	6.28	1.19	1845.76
RS2	pRSC3	GU391955	Cb	2.83	7.89	9.46	7.98	10.95	4.98	122.12
RS3	pRSC4	GU391956	Cb	2.11	1.29	5.17	6.19	7.36	1.32	2702.35
RS4	pRSC5	GU391957	Cb	2.50	5.78	10.56	4.86	4.38	4.11	5113.16
RS5	pRSC6	GU391958	Cb	3.07	5.21	6.73	6.11	35.51	3.78	152.22
RS6	pRSC7	GU391959	Cb	1.88	25.99	22.16	5.50	31.34	5.24	1370.04
RS7	pRSC8	GU391960	Cb	18.13	16.45	1.18	155.42	137.19	28.44	2194.99
RS8	pRSC10	GU391962	Cb	14.72	10.27	17.63	17.39	21.56	1.13	317.37
RS9	pRSC11	GU391963	Cb	1.15	1.25	2.14	3.97	-	-	2817.11
RS10	pRSC12	GU391964	Cb	22.63	68.59	68.12	69.07	92.41	33.36	4705.07
RS11	pRSC13	GU391965	Cb	35.26	280.14	284.05	44.63	560.28	227.54	410.15
RS12	pRSC14	GU391966	Cb	7.84	29.04	168.90	259.57	24.08	11.16	916.51

The Cb represents the calibrator tissue showing least expression (expression value 1) with which the comparisons for the expression in other tissues were made. The value in each row signifies the corresponding fold of expression which is higher as compared to the calibrator value.

### Full length CDS of the*TSPY1*-like and *COL6A1* genes

Using 3′ RACE, we obtained the 1222 bp cDNA sequence of the *TSPY1*-like gene [GenBank: HQ104923], which has 975 bp of protein coding sequence. The amino acid sequence of the TSPY1-like protein was derived *in silico* from the Transeq Nucleotide to Protein Sequence Conversion/EMBOSSES Transeq/EBI [41]. A nucleotide blast of the water buffalo *TSPY1*-like gene found that it had>90% identity with *Bos taurus TSPY1*, which is consistent with the results from a protein blast (Figure S1). However, the water buffalo cDNA sequence of *TSPY1* showed approximately 40–60% identity with other mammalian species.

**Figure 6:**
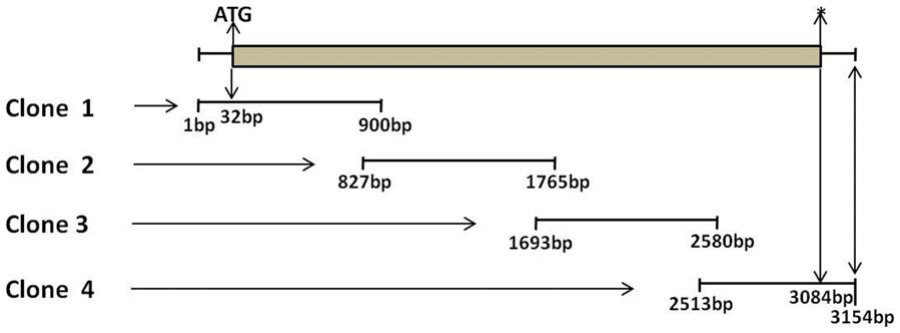
Schematic illustration showing the cloning strategy for the water buffalo *COL6A1* gene. Different overlapping clones of the water buffalo *COL6A1* gene were generated to obtain the full length cds. Shown here are the nucleotide boundaries and positions of start and stop codons.

Full length 3154 bp cds of the *COL6A1* gene [GenBank: HQ104922] was obtained from the assembly of four clones (Figure 6). The *COL6A1* gene of *Bubalus bubalis* has 3084 bp of protein coding sequence. The amino acid sequence of the COL6A1 protein was derived *in silico*. This gene seems to be conserved across the mammalian species showing a very high percent identity between cattle and water buffalo (98% with *Bos taurus COL6A1*). However, the water buffalo *COL6A1* gene showed sequence identity in the range of 73–90% with other mammals (Figure S2).

### Chromosomal localization and copy number status of the *TSPY1-like* gene

**Figure 7:**
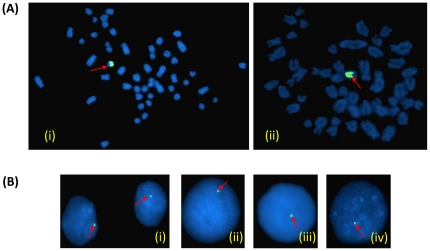
Chromosomal localization of *TSPY1*-*like* gene. The*TSPY1-*like gene probe (arrows) localized to the water buffalo metaphase chromosome Y (**A**) (i and ii) and interphase nuclei (**B**) (i–iv).

**Figure 8:**
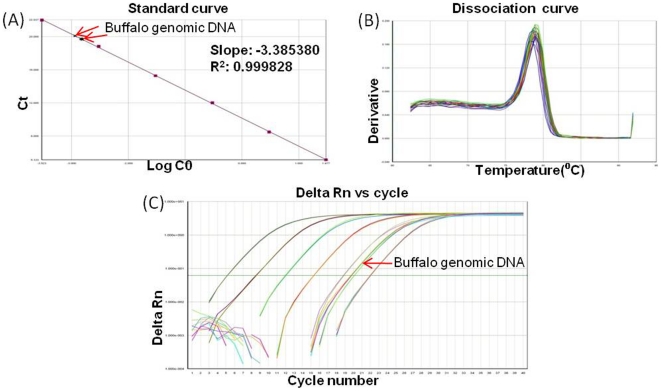
Copy number assessment of *TSPY1*-*like* gene by q-PCR. q-PCR amplification plot from a 10-fold serial dilution of the plasmid for copy number calculation. (**A**) Standard curve, (**B**) Dissociation curve showing single peak, which indicates primer specificity with the target DNA. (**C**) Delta Rn vs Cycle showing amplification plots of the standard plasmid and water buffalo genomic DNA.

FISH of the *TSPY1*-like gene using the bovine BAC probe CH240-127C20 [GenBank: AC234853.4] resulted in signals on the water buffalo Y chromosome (Figure 7). Based on the Q-PCR, the copy number of this gene was found to vary from 182–202 per genome (Figure 8).

## Discussion

Satellite sequences have attracted a great deal of attention due to their involvement in gene regulation and genomic imprinting [11], [42]–[45]. We identified 53 transcripts tagged with the trimeric repeat (TGG)_n_ in the water buffalo genome, which seems to be the first such study in any animal system. This is significant because TGG repeats, owing to their shrinkage and expansion, affect gene expression and are implicated in human diseases [12], [46]. Differential expression of these transcripts across the tissues sampled and dramatically high expression of 11 in the spermatozoa suggests that a set of these genes are reserved for testicular functions. It was not possible for us to molecularly characterize all 53 genes in this study; we therefore focused on two genes, *TSPY1* and *COL6A1*. *TSPY1* was found to be testis-specific by RT-PCR corroborating earlier studies [47], [48], whereas *COL6A1* showed ubiquitous expression. In the present study, we did not depend on RT-PCR data for assessing the level of expression of a gene and instead conducted q-PCR. Thus, the expression levels detected by q-PCR could be used to support a tissue-specific function of a gene.

The *TSPY1*-like sequence amplified from *Bubalus bubalis* showed >95% identity with that of *Bos taurus* [GenBank: XM_001254382.2] and the BAC clone of *Bos taurus* Y Chromosome CH240-127C20 [GenBank: AC234853.4]. In other species, this gene showed approximately 50% identity and a high level of heterogeneity. Invariably, this gene is referred to as *TSPY1*-like gene. We propose that the same may be referred to as *TSPY1* because we have detected its high level of expression in testes and localized it to the water buffalo Y chromosome. There is a remarkable degree of variation in the copy number of this gene among different mammalian species. The rat has one pseudo and one functional copy while the mouse has only one pseudo gene [49], [50]. Recent study of the different breeds of *Bos taurus* confirms gross variation in the copy number of this gene [51]. Significantly, an increase in copy number of *tspy* in humans is linked with male infertility [52], while a decrease is associated with prostate cancer [53]. The copy number of *tspy* ranges 20–60 in human males and 50–200 in bovid males [54], [55], which is consistent with our present study. More copies are associated with an enhanced level of protein synthesis [56]. However, it is not clear if more copies of this gene protect a human male from prostate cancer. Even if they do, a high copy number of this gene in humans is associated with infertility [52]. Taken together, we purpose that a critical balance of the copy number of *TSPY1* is maintained across the mammalian species. Arguably, the high copy number may act as a compensatory strategy against the decay or loss of other genes involved in fertility [57]. It would be of relevance to undertake a detailed study on the copy number variation of this gene amongst different categories of infertility in both human and animal males to resolve this issue. This would determine if a greater number of copies are indeed associated with infertility.

The transcripts (having significant identity of up to >90%) uncovered by MASA were studied further to determine the family of proteins to which they belong using the conserved domain database of NCBI [58]. The results suggest that these repeats are not specific to genes belonging to a particular protein family and are instead distributed throughout the coding genome.

*TSPY* (Testis specific protein, Y-encoded), a member of the greater SET (Su(var)3–9, Enhancer-of-zeste, Trithorax)/NAP (Nucleosome assembly protein) family of molecules, has been implicated in the regulation of gene expression, malignant development of gonadoblastoma and testicular and prostate cancer [59], [60]. These proteins are involved in nucleosome assembly, chromatin fluidity and trafficking histones into the nucleus [61]–[63].

The *COL6A1* gene encodes the alpha-1 subunit of type VI collagen and belongs to the vWFA (von Willebrand factor (vWF) type A) superfamily. Collagens are involved in the formation of the fibrillar and microfibrillar networks of the extracellular matrix, basement membranes and other structures of the extracellular matrix. Some collagens contain approximately 15–18 vWA domains. The vWA domains of extracellular eukaryotic proteins mediate adhesion via metal ion-dependent adhesion sites (MIDAS) [64]. Mutations in any one of these genes that code for collagen VI subunits results in the autosomal dominant disorder Bethlem myopathy and Ullrich scleroatonic muscular dystrophy [65]. Our MASA-based approach enabled us to identify genes in animal systems that are known to have clinical significance.

Earlier, using decoy oligo primers based on GACA/GATA [37], with a consensus of 33.15 [35] and 33.6 repeat loci [40], we demonstrated an association between a large number of mRNA transcripts and these repeat elements in water buffalo. The genes tagged with these STRs are likely favored evolutionarily. Accordingly, we also studied satellite tagged mRNA transcripts in spermatozoa. A number of signaling molecules and transcription factors have been reported to be both present in spermatozoa and transported into the zygotic cytoplasm [23], [25], [27]. The presence of TGG tagged transcripts that are most highly expressed in spermatozoa and the testes adds to this finding.

### Conclusion

The water buffalo has several recognized and undocumented breeds of which a few are considered to be superior livestock and belong to elite categories. However, the genetic basis of their superiority is not yet established. Present work demonstrates that the trimeric repeats (TGG) is present in a number of functional genes of the water buffalo that show tissue-specific expression. Genes showing high levels of expression in the testes and spermatozoa are potential candidates for in-depth characterization in both normal and genetically infertile animals. In-depth analysis of such genes is hoped to focus the search for the elusive ones that confer desired characteristics to livestock. This would add a new dimension to genome analysis and augment animal biotechnology.

## Supporting Information

Figure S1 **Nucleotide sequence alignment (i) and amino acid sequence (ii) of the** ***TSPY1*** **-like gene of water buffalo and cattle.** Water buffalo and cattle show ∼95% identity at the nucleotide level.
(DOC)

Figure S2 **Multiple alignment of the** ***COL6A1*** **gene (i), phylogenetic tree based on nucleotide sequence (ii), multiple alignment of amino acid sequence of the** ***COL6A1*** **protein (iii) and phylogenetic tree based on amino acid sequence (iv) of different species.** Note the close relationship between cattle and water buffalo in the phylogenetic tree. Horse, as expected, has a distant relationship with water buffalo and cattle, whereas mouse and rat group together.
(DOC)

Table S1 **List of primers used to test for genomic DNA contamination in the samples.** The primers for *ACTB* were designed in our lab, while those for *CD45* and *CDH1* genes were based on an earlier report [31]. Primers corresponding to *CD45* and *CDH1* span several introns but their positions were not defined.
(DOC)

Table S2 **Detailed analysis of MASA identified somatic, gonadal and spermatozoal mRNA transcripts tagged with the TGG repeat from the water buffalo** ***Bubalus bubalis.*** (**i**), Transcripts identified from the somatic and gonadal tissues. (**ii**), Transcripts identified from spermatozoa. All transcripts, their accession number and their homology status are listed in this table.
(DOC)

Table S3 **List of primers used for RT-PCR on cDNA from different tissues (i) and semen (ii).** The primer IDs and corresponding gene accession number of the amplified transcripts are given in the table.
(DOC)

Table S4 **List of primers used for q-PCR on cDNA from different tissues (i) and semen samples (ii).**
(DOC)

Table S5 **List of primers used for obtaining full length CDS of the** ***COL6A1*** **gene (i) and** ***TSPY1*** **-like gene (ii).**
(DOC)

Table S6 **Details of the clones corresponding to different mRNA transcripts uncovered by MASA showing significant homologies with the cDNA sequences in the Database.** This table shows details of only 14 mRNA transcripts of the 38 identified from different somatic tissues and gonads. Significantly, all the query sequences, irrespective of their size, showed >95% identity with the sequences in the GenBank.
(DOC)
