# Supplementary material for: Molecular Mining of Alleles in Water Buffalo Bubalus bubalis and Characterization of the TSPY1 and COL6A1 Genes
Source: PLoS One. 2011 Sep 15;6(9):e24958. doi: 10.1371/journal.pone.0024958 (PMC3174239; doi:10.1371/journal.pone.0024958)
Supplement: Figure S1 — Nucleotide sequence alignment (i) and amino acid sequence (ii) of the TSPY1 -like gene of water buffalo and cattle. Water buffalo and cattle show ∼95% identity at the nucleotide level. (DOC) [file pone.0024958.s001.doc]

**Figure S1:**

**(i) Nucleotide sequence alignment of *TSPY1-*like mRNA of Buffalo *Bubalus bubalis* and Cattle *Bos tauras***

Buffalo ATGGAGAGTGAGACGGGGCCAGAGGAAGGCGGCAGCACTCCGGGATCCTGGACCTTGGTT 60

Cattle ATGGAGAGTGAGACGGGGCCAGAGGAAGGCGGCAGCACTCCGGGATCCTGGATCTTAGTT 60

**************************************************** *** ***

Buffalo GTGAGCCCGGGTCTTCACGAGGGAGGGGCCCTGGGGCCTAGCAGCCCGGTGGGGGCGGCA 120

Cattle GTGAGCCCGGGTCTTCATGAGGGAGGGGCCCTGGGGCCTACCAGCTCCGTGTGGGCGGCA 120

***************** ********************** **** * *** ********

Buffalo GAGGCGATGCAGGCCGCAGGTGGCGCGCCAGGCGAGGAGGCCGCCCTCTTCTGGGTGGAG 180

Cattle GAGGCGATGCAGGCCGCAGGTGGTGCGCCAGGCGAGGAGGCCGCCCTCTTCTGGGTGGAG 180

*********************** ************************************

Buffalo GCAGTGGAGGAAAGTGCGGCCCTGGAGGAGGGAGAGGTGGCGGGAATCGGGCAGGAGTTC 240

Cattle GCAGTGGAGGAAGGTGCGGCTGTGGAGGAGGGAGAGGTGGCGGGACCCGGGCAGGAGTTC 240

************ ******* *********************** *************

Buffalo CAGCTGCTGGTGTTGGACGTCATGGAGGAGGTGGAGGTGGTGGCATACGAGGAGCAGGAG 300

Cattle CAGCTGCTGGTGTTGGACGTCATGGAGGAGGTGGAGGTGGTGGCATACGAGGAGCAGGAG 300

************************************************************

Buffalo CAGGTGTCCTCGGAGGAGCCTGTCCACGAACATCCAAGGCCCGGCGCCCCGAGTGACCGG 360

Cattle CAGGTGTCCTCGGAGGAGCATGTCCACGACCATCCAAGGCCCGGAGCCCTGAGTGACCGG 360

******************* ********* ************** **** **********

Buffalo CCTGCACTGGAGGCGCTGGCGGCCCTGCAGCTGGAGCTGGAGCCCGTGAATAAGGAAGCC 420

Cattle CCTGCACTGGAGGCGCTGGCGGCCCTGCAGCTGGAGCTGGAGCCCGTGAATCAGAAAGCC 420

*************************************************** ** *****

Buffalo CAAAGGGCGCATGCGCGCCTGAAACGTAAGACCTGTCAGCGGCGGAAGCTGCATCTGGAA 480

Cattle CAAAGGGCGCATGCTCGCCTGAAACATAAGACCAGTCAGCGGCGGAAGGTGCATCTAGAA 480

************** ********** ******* ************** ******* ***

Buffalo CACAGAAGCGCCATCATCCAGGGCATCCATGGCTTCTGGGTCGAGGTTTTTATGAACCAC 540

Cattle CACAGAAGCGCCATCATCCAGGGCATCCGTGGCTTCTGGGTCGAAGTTTTTATGAACCAC 540

**************************** *************** ***************

Buffalo CCCCAAATGTCAGTTTTGATGAGCAAGCAAGATGCAGACATGCTTCACTTCATGACCAAC 600

Cattle CCCCAAATGTCAGTTTTGATGAGCAAGCAAGATGCAGACATGCTTCACTTCATGACCAAC 600

************************************************************

Buffalo TTGAAGGTGGAGGAATTCAGGCATCCCACTCATCACTGCAAGATCACATTGTCCTTTCGG 660

Cattle TTGGAGGTGGAGGAATTCAGGCATCCCACTCGTCACTGCAAGATCACATTGTCCTTTCGG 660

*** *************************** ****************************

Buffalo AGGAATAGGTATTTCCAGGATGAAGTGATTGTCAAGGAGTACCTGATTAAGGTTACTGGA 720

Cattle AGGAATAGGTATTTCCAGAATGAAGTGATTGTCAAGGAGTACCTGATGAAGGTCACTGGA 720

****************** **************************** ***** ******

Buffalo TACCAAGCATCTCGTTCCACTCCAGTTCAGTGGTACCAGGGCTTTGAACGGAAGGCATAC 780

Cattle TACCACGCATCTCGTTCCACTCCAGTTCAGTGGCACCAGGGCTTTGAATGGAAGGCATAC 780

***** *************************** ************** ***********

Buffalo AGGCGCAGGCACCACGACAGCAGCGTTAACTTCTTCAACTGGTTCTTTGACCACAACTTC 840

Cattle AGGCGCAGGCACCACGACAGCAGCGTTAACTTCTTCAACTGGTTCTTTGACCACAATTTC 840

******************************************************** ***

Buffalo ACAGGATCTGACAGGATTGCTGAGATCATCATAAAGGACCTGTGGCCCAATCCTTTGCAG 900

Cattle ACAGGATCTGACTGGATTGCTGAGATCATCATAAGGGATCTGTGGCCCAATCCTTTGCAG 900

************ ********************* *** *********************

Buffalo TACTACGTGAGGAGGAAGGCTGCACCACGGGAGGTACCAGGAGGACGAGAGG---AACCC 957

Cattle TACTATGTGAGGAGGAAGGCTGCACCACAAAAGGTACCAGGAGGACGAGAGGTGAGGCGC 960

***** ********************** ********************* * *

Buffalo CCTCCCCCCAGCTTTTGA------------------------------------------ 975

Cattle CCAGGGGCCGAGCACTGGCATAAGTGTTGTCAAAAACTTGGGTCATTTATTCCACTTGTG 1020

** ** **

Buffalo ---------------------------------------------

Cattle AAGACAGTGAGACTCCGTGGAGTACAGAGTGACACCAGGGCATGA 1065

**(ii) Amino acid sequence alignment of TSPY1-like protein of Cattle *Bos tauras* and Buffalo *Bubalus bubalis***

Cattle MESETGPEEGGSTPGSWILVVSPGLHEGGALGPTSSVWAAEAMQAAGGAPGEEAALFWVE 60

Buffalo MESETGPEEGGSTPGSWTLVVSPGLHEGGALGPSSPVGAAEAMQAAGGAPGEEAALFWVE 60

***************** ***************:*.* **********************

Cattle AVEEGAAVEEGEVAGPGQEFQLLVLDVMEEVEVVAYEEQEQVSSEEHVHDHPRPGALSDR 120

Buffalo AVEESAALEEGEVAGIGQEFQLLVLDVMEEVEVVAYEEQEQVSSEEPVHEHPRPGAPSDR 120

****.**:******* ****************************** **:****** ***

Cattle PALEALAALQLELEPVNQKAQRAHARLKHKTSQRRKVHLEHRSAIIQGIRGFWVEVFMNH 180

Buffalo PALEALAALQLELEPVNKEAQRAHARLKRKTCQRRKLHLEHRSAIIQGIHGFWVEVFMNH 180

*****************::*********:**.****:************:**********

Cattle PQMSVLMSKQDADMLHFMTNLEVEEFRHPTRHCKITLSFRRNRYFQNEVIVKEYLMKVTG 240

Buffalo PQMSVLMSKQDADMLHFMTNLKVEEFRHPTHHCKITLSFRRNRYFQDEVIVKEYLIKVTG 240

*********************:********:***************:********:****

Cattle YHASRSTPVQWHQGFEWKAYRRRHHDSSVNFFNWFFDHNFTGSDWIAEIIIRDLWPNPLQ 300

Buffalo YQASRSTPVQWYQGFERKAYRRRHHDSSVNFFNWFFDHNFTGSDRIAEIIIKDLWPNPLQ 300

*:*********:**** *************************** ******:********

Cattle YYVRRKAAPQKVPGGREVRRPGAEHWHKCCQKLGSFIPLVKTVRLRGVQSDTRA 354

Buffalo YYVRRKAAPREVPGGREEPPP------------PSF------------------ 324

*********::******
