# Supplementary material for: Molecular Mining of Alleles in Water Buffalo Bubalus bubalis and Characterization of the TSPY1 and COL6A1 Genes
Source: PLoS One. 2011 Sep 15;6(9):e24958. doi: 10.1371/journal.pone.0024958 (PMC3174239; doi:10.1371/journal.pone.0024958)

**Figure S2:**

**(i) Multiple sequence alignment of *COL6A1* mRNA of different organisms**

Buffalo ---------------------------ATGAGGCTGCCCCGCGCTCTGCTCCCCCTGCTG 33

Cattle ---------------------------ATGAGGCTGCCCCGCGCTCTGCTCCCCCTGCTG 33

Horse ---------------------------ATGAGGCTGGCCAGCGCTCTGCTCCTGCTGCTG 33

Human ---------------------------ATGAGGGCGGCCCGTGCTCTGCTGCCCCTGCTG 33

Chimpanzee ---------------------------ATGAGGGCGGCCCGTGCTCTGCTGCCCCTGCTG 33

Mouse ---------------------------ATGAGGCTGGCCCACGCTCTGCTGCCCCTGCTG 33

Rat ATGGTGACTCGAAGGCCAGCCGGAAACATGAGGCTGGCCCACACTCTGCTGCCCCTGCTG 60

****** * ** ******* * ******

Buffalo CTGCAGGCCTGCTGGGCCTCCGCGCAGGACGACACCGTGGCCTCAAGGGCCATCGCCTTC 93

Cattle CTGCAGGCCTGCTGGGCCTCCGCGCAGGACGACCCCGTGGCCTCAAGGGCCATCGCCTTC 93

Horse CTGCAGGCCTGCTGGGCCGCTGCACAGGATGACACGGCCGCCGTGAGGACCGTCGCCTTC 93

Human CTGCAGGCCTGCTGGACAGCCGCGCAGGATGAGCCGGAGACCCCGAGGGCCGTGGCCTTC 93

Chimpanzee CTGCAGGCCTGCTGGACAGCCGCGCAGGATGAGCCGGAGACCCCGAGGGCCGTGGCCTTC 93

Mouse CTACAAGCCTGCTGGGTGGCCACACAGGACATCC---AGGGCTCCAAAGCGATTGCCTTC 90

Rat CTACAGGCCTGCTGGGTGGCCGCACAGGACATCC---AGGGCTCTAGAGCGATTGCCTTC 117

** ** ********* * * ***** * * * * ******

Buffalo CAAGACTGCCCTGTGGACCTGTTCTTTGTGCTGGACACCTCCGAGAGTGTGGCCTTGAGG 153

Cattle CAAGACTGCCCTGTGGACCTGTTCTTTGTGCTGGACACCTCCGAGAGCGTGGCCTTGAGG 153

Horse CAAGACTGCCCCGTGGACCTGTTCTTCGTGCTGGACACCTCCGAGAGCGTGGCCCTGAGG 153

Human CAGGACTGCCCCGTGGACCTGTTCTTTGTGCTGGACACCTCTGAGAGCGTGGCCCTGAGG 153

Chimpanzee CAGGACTGCCCCGTGGACCTGTTCTTTGTGCTGGACACCTCTGAGAGTGTGGCCCTGAGG 153

Mouse CAAGACTGCCCTGTGGATCTATTCTTCGTGCTCGACACCTCGGAGAGTGTGGCCTTGAGG 150

Rat CAAGACTGCCCTGTGGATCTGTTCTTCGTGCTGGACACCTCGGAGAGTGTGGCCTTGAGG 177

** ******** ***** ** ***** ***** ******** ***** ****** *****

Buffalo CTGAAGCCCTATGGGGCCCTGGTGGACAAGGTCAAGTCCTTCACCAAGCGCTTCATTGAC 213

Cattle CTGAAGCCCTATGGGGCCCTGGTGGACAAGGTCAAGTCCTTCACCAAGCGCTTCATTGAC 213

Horse CTGAAACCCTACGGGGCCCTGGTGGACAAGGTCAAGGCCTTCACCAAGCGCTTCATCGAC 213

Human CTGAAGCCCTACGGGGCCCTCGTGGACAAAGTCAAGTCCTTCACCAAGCGCTTCATCGAC 213

Chimpanzee CTGAAGCCCTACGGGGCCCTCGTGGACAAAGTTAAGTCCTTCACCAAGCGCTTCATCGAC 213

Mouse CTGAAACCTTATGGGGCCTTGGTGGACAAGGTGAAGTCCTTCACTAAGCGCTTCATTGAC 210

Rat CTGAAACCTTATGGGGCCCTGGTGGACAAGGTGAAATCCTTTACCAAGCGTTTCATCGAC 237

***** ** ** ****** * ******** ** ** **** ** ***** ***** ***

Buffalo AACCTGAAGGACAGGTACTACCGCTGTGACCGCAACCTGGTGTGGAATGCGGGAGCGCTG 273

Cattle AACCTGAACGACAGGTACTACCGCTGTGACCGCAACCTGGTGTGGAATGCGGGCGCGCTG 273

Horse AACCTGAGGGACAGGTACTACCGGTGTGACCGAAACCTGGTGTGGAATGCTGGCGCGCTG 273

Human AACCTGAGGGACAGGTACTACCGCTGTGACCGAAACCTGGTGTGGAACGCAGGCGCGCTG 273

Chimpanzee AACCTGAGGGACAGGTACTACCGCTGTGACCGAAACCTGGTGTGGAACGCAGGCGCGCTG 273

Mouse AACCTGAGAGACAGGTACTACCGGTGTGACCGCAACCTGGTTTGGAATGCGGGTGCGCTG 270

Rat AACCTGAGAGACAGGTACTACCGATGTGACCGCAACCTGGTTTGGAATGCGGGCGCGCTG 297

******* ************** ******** ******** ***** ** ** ******

Buffalo CACTATAGCGACGAGGTGGAGATCATCCGTGGGCTCACGCGCATGCCCAGCGGCCGCGAC 333

Cattle CACTATAGCGACGAAGTGGAGATCATCCGCGGGCTCACGCGCATGCCCAGCGGCCGCGAC 333

Horse CACTACAGTGATGAGGTGGAGATCATCCGTGGGCTCACGCGCATGCCCAGTGGCCGCGAC 333

Human CACTACAGTGACGAGGTGGAGATCATCCAAGGCCTCACGCGCATGCCTGGCGGCCGCGAC 333

Chimpanzee CACTACAGTGACGAGGTGGAGATCATCCAAGGCCTCACGCGCATGCCTGGCGGCCGCGAC 333

Mouse CACTACAGTGACGAGGTGGAGATCATCCGAGGGCTCACGCGCATGCCCAGTGGCCGCGAT 330

Rat CACTACAGTGACGAGGTGGAGATCATCAGAGGGCTCATGCGCATGCCCAGTGGCCGTGAC 357

***** ** ** ** ************ ** **** ********* * ***** **

Buffalo GAGCTTAAGAGCAGCGTGGACGCTGTCAAGTACTTCGGAAAGGGCACCTATACCGACTGC 393

Cattle GAGCTTAAGAGCAGCGTGGACGCCGTCAAGTACTTCGGAAAGGGCACCTATACCGACTGC 393

Horse GAGCTCAAGGCCAGCGTGGATGCGGTCAAGTACTTCGGCAAGGGCACCTACACAGACTGC 393

Human GCACTCAAAAGCAGCGTGGACGCGGTCAAGTACTTTGGGAAGGGCACCTACACCGACTGC 393

Chimpanzee GCACTCAAAAGCAGCGTGGACGCGGTCAAGTACTTTGGGAAGGGCACCTACACCGACTGT 393

Mouse GAGCTCAAGGCCAGCGTGGATGCGGTCAAGTACTTCGGGAAAGGCACCTACACCGACTGC 390

Rat GAGCTCAAAGCCAGCATAGATGCGGTCAAGTACTTCGGGAAAGGCACCTACACCGACTGT 417

* ** ** **** * ** ** *********** ** ** ******** ** *****

Buffalo GCCATCAAGAAGGGTCTGGAGGAGCTGCTCGTGGGGGGCTCCCACCTGAAGGAGAACAAG 453

Cattle GCCATCAAGAAGGGTCTGGAGGAGCTGCTCGTGGGGGGCTCCCACCTAAAGGAGAACAAG 453

Horse GCCATCAAGAAGGGGCTGGAGGAGCTGCTCGTGGGTGGCTCCCACCTGAAGGAGAACAAG 453

Human GCTATCAAGAAGGGGCTGGAGCAGCTCCTCGTGGGGGGCTCCCACCTGAAGGAGAATAAG 453

Chimpanzee GCCATCAAGAAGGGGCTGGAGCAGCTCCTCGTGGGGGGCTCCCACCTGAAGGAGAATAAG 453

Mouse GCCATTAAGAAGGGGCTGGAGGAGCTGCTCATAGGGGGCTCCCACCTGAAGGAGAACAAG 450

Rat GCCATTAAGAAGGGGCTGGAGGAGCTGCTCATTGGGGGCTCCCACCTAAAAGAGAACAAG 477

** ** ******** ****** **** *** * ** *********** ** ***** ***

Buffalo TACCTGGTCGTGGTGACTGACGGGCACCCCCTGGAGGGCTACAAGGAGCCCTGCGGGGGC 513

Cattle TACTTGGTTGTGGTGACTGACGGGCACCCCCTGGAGGGCTACAAGGAGCCCTGCGGGGGC 513

Horse TACCTGATCGTGGTGACCGACGGGCACCCCCTCGAGGGCTACAAGGAGCCATGCGGGGGC 513

Human TACCTGATTGTGGTGACCGACGGGCACCCCCTGGAGGGCTACAAGGAACCCTGTGGGGGG 513

Chimpanzee TACCTGATTGTGGTGACCGACGGGCACCCCCTGGAGGGCTACAAGGAACCCTGTGGGGGG 513

Mouse TACTTGATCGTGGTGACCGACGGGCATCCTCTAGAGGGCTACAAGGAACCATGCGGGGGT 510

Rat TACTTGATCGTGGTGACTGACGGGCATCCCCTCGAGGGCTACAAGGAACCATGTGGGGGT 537

*** ** * ******** ******** ** ** ************** ** ** *****

Buffalo CTGGAGGATGCTGTGAACGAGGCCAAGCATCTGGGCATCAAAGTCTTCTCCGTGGCCATC 573

Cattle CTGGAGGATGCTGTGAACGAGGCCAAGCATCTGGGCATCAAAGTCTTCTCCGTGGCCATC 573

Horse CTGGAGGACGCCGTCAACGAGGCCAAGCACCTGGGCATCAAAGTCTTCTCCGTGGCCATC 573

Human CTGGAGGATGCTGTGAACGAGGCCAAGCACCTGGGCGTCAAAGTCTTCTCGGTGGCCATC 573

Chimpanzee CTGGAGGATGCTGTGAACGAGGCCAAGCACCTGGGCGTCAAAGTCTTCTCGGTGGCCATC 573

Mouse CTGGAAGATGCAGTAAATGAGGCCAAACACCTGGGCATCAAGGTCTTTTCTGTGGCCATC 570

Rat CTGGAAGATGCAGTGAACGAGGCCAAACACTTGGGCATCAAGGTCTTTTCTGTGGCCATC 597

***** ** ** ** ** ******** ** ***** **** ***** ** *********

Buffalo ACGCCCGACCACCTGGAGCCACGTCTGAGCATCATCGCCACGGACCACACGTACCGGCGC 633

Cattle ACGCCCGACCACCTGGAGCCACGTCTGAGCATCATCGCCACGGACCACACGTACCGGCGC 633

Horse ACGCCTGACCACCTGGAGCCACGTCTGAGCATCATTGCCACGGACCACACGTACCGGCGC 633

Human ACACCCGACCACCTGGAGCCGCGTCTGAGCATCATCGCCACGGACCACACGTACCGGCGC 633

Chimpanzee ACACCCGACCACCTGGAGCCGCGTCTGAGCATCATCGCCACGGACCACACGTACCGGCGC 633

Mouse ACACCTGACCACCTGGAGCCACGTCTAAGTATCATTGCCACAGACCACACATACCGGCGC 630

Rat ACACCTGATCACCTGGAGCCACGTCTAAGCATCATCGCCACAGACCACACATACCGGCGC 657

** ** ** *********** ***** ** ***** ***** ******** *********

Buffalo AACTTCACGGCGGCCGACTGGGGGCAGAGCCGCGACGCCGAGGAAGTCATCAGCCAGACC 693

Cattle AACTTCACGGCGGCTGACTGGGGGCAGAGCCGCGACGCCGAGGAAGTCATCAGCCAGACC 693

Horse AACTTCACGGCGGCCGACTGGGGGCAGAGCCGCGACGCAGAAGAGATCATCAGCCAGACC 693

Human AACTTCACGGCGGCTGACTGGGGCCAGAGCCGCGACGCAGAGGAGGCCATCAGCCAGACC 693

Chimpanzee AACTTCACGGCGGCTGACTGGGGCCAGAGCCGCGACGCAGAGGAGGCCATCAGCCAGACC 693

Mouse AATTTCACGGCAGCTGACTGGGGGCATAGCCGCGATGCAGAAGAGGTCATCAGCCAGACC 690

Rat AATTTCACGGCTGCTGACTGGGGGCATAGTCGAGATGCGGAAGAGACCATCAGCCAGACC 717

** ******** ** ******** ** ** ** ** ** ** ** *************

Buffalo ATTGAAACCATCACGGACATGATCAAAAATAATGTGGAGCAAGTGTGCTGCTCCTTCGAG 753

Cattle ATCGACACCATCACCGACATGATCAAAAATAATGTGGAGCAAGTGTGCTGCTCCTTCGAG 753

Horse ATCGACACCATCACCGACATGATCAAAAACAATGTGGAGCAAGTGTGCTGCTCCTTCGAG 753

Human ATCGACACCATCGTGGACATGATCAAAAATAACGTGGAGCAAGTGTGCTGCTCCTTCGAA 753

Chimpanzee ATCGACACCATCGTGGACATGATCAAAAATAACGTGGAGCAAGTGTGCTGCTCCTTCGAA 753

Mouse ATTGACACCATTGTGGACATGATTAAAAATAACGTGGAACAAGTGTGTTGTTCTTTTGAG 750

Rat ATTGACACCATTGTGGACATGATTAAAAATAATGTGGAGCAAGTGTGTTGTACTTTTGAG 777

** ** ***** ******** ***** ** ***** ******** ** * ** **

Buffalo TGCCAGCCCGCCAGAGGACCCCCCGGGCCTCGAGGCGACCCTGGGTACGAGGGAGAAAGA 813

Cattle TGCCAGCCTGCCAGAGGACCCCCCGGGCCTCGAGGCGACCCCGGGTACGAGGGAGAAAGA 813

Horse TGTCAGCCCGCCAGAGGACCCCCAGGCCTGCGGGGCGACCCCGGGTATGAGGGAGAACGA 813

Human TGCCAGCCTGCAAGAGGACCTCCGGGGCTCCGGGGCGACCCCGGCTTTGAGGGAGAACGA 813

Chimpanzee TGCCAGCCTGCAAGAGGACCTCCGGGGCTCCGGGGCGACCCCGGCTTTGAGGGAGAACGA 813

Mouse TGCCAGGCTGCCAGAGGACCTCCAGGGCCCCGAGGCGACCCTGGGTATGAGGGGGAGCGA 810

Rat TGCCAGGCTGCCAGAGGACCTCCAGGGCCCCGGGGCGACCCTGGGTATGAGGGGGAACGA 837

** *** * ** ******** ** ** * ** ******** ** * ***** ** **

Buffalo GGGAAGCCGGGGCTCCCAGGAGAGAAAGGAGAAGCTGGAGACCCTGGAAGACCTGGGGAC 873

Cattle GGGAAGCCGGGGCTCCCGGGAGAGAAAGGAGAAGCTGGAGACCCTGGAAGACCTGGGGAC 873

Horse GGGAAGCCGGGTCTGCCTGGAGAGAAAGGAGAAGCCGGAGACCCCGGAAGGCCCGGGGAC 873

Human GGCAAGCCGGGGCTCCCAGGAGAGAAGGGAGAAGCCGGAGATCCTGGAAGACCCGGGGAC 873

Chimpanzee GGCAAGCCGGGGCTCCCAGGAGAGAAGGGAGAAGCCGGAGATCCTGGAAGACCCGGGGAC 873

Mouse GGAAAGCCAGGTCTTCCGGGAGAGAAGGGAGAAGCTGGAGACCCTGGACGACCTGGGGAT 870

Rat GGAAAGCCGGGTCTTCCCGGAGAGAAGGGAGAAGCTGGAGACCCTGGACGACCTGGGGAT 897

** ***** ** ** ** ******** ******** ***** ** *** * ** *****

Buffalo CTTGGACCCGTTGGCTACCAGGGCATGAAGGGAGAAAAAGGGAGCCGAGGGGAGAAGGGC 933

Cattle CTTGGACCCGTCGGCTACCAGGGCATGAAGGGAGAAAAAGGGAGCCGAGGGGAGAAGGGC 933

Horse CTCGGACCCGTTGGCTACCAGGGGATGAAGGGAGAAAAAGGGAGCCGAGGGGACAAGGGC 933

Human CTCGGACCTGTTGGGTACCAGGGAATGAAGGGAGAAAAAGGGAGCCGTGGGGAGAAGGGC 933

Chimpanzee CTCGGACCTGTTGGGTACCAGGGGATGAAGGGAGAAAAAGGGAGCCGTGGGGAGAAGGGC 933

Mouse CTTGGACCAGTCGGGTACCAGGGTATGAAGGGAGAAAAGGGGAGCCGTGGAGAGAAGGGT 930

Rat CTTGGACCAGTCGGGTACCAGGGTATGAAGGGAGAAAAGGGAAGCCGTGGAGAGAAGGGT 957

** ***** ** ** ******** ************** ** ***** ** ** *****

Buffalo TCCAGGGGACCCAAAGGCTACAAGGGCGAGAAGGGAAAGCGTGGCATGGACGGCGTGGAT 993

Cattle TCCAGGGGACCCAAAGGCTACAAGGGCGAGAAGGGAAAGCGTGGCATGGACGGCGTGGAT 993

Horse TCCAGGGGACCCAAGGGCTACAAGGGTGAGAAGGGCAAGCGCGGCATCGACGGTGTGGAT 993

Human TCCAGGGGACCCAAGGGCTACAAGGGAGAGAAGGGCAAGCGTGGCATCGACGGGGTGGAC 993

Chimpanzee TCCAGGGGACCCAAGGGCTACAAGGGAGAGAAGGGCAAGCGTGGCATCGACGGGGTGGAT 993

Mouse TCCAGAGGACCGAAAGGTTACAAGGGCGAGAAAGGCAAGCGCGGAATCGACGGGGTCGAC 990

Rat TCCAGAGGCCCGAAAGGCTACAAGGGCGAGAAAGGCAAGCGCGGCATCGATGGGGTGGAC 1017

***** ** ** ** ** ******** ***** ** ***** ** ** ** ** ** **

Buffalo GGCATGAAGGGGGAGACAGGGTACCCTGGCCTGCCAGGCTGCAAGGGCTCACCCGGATTC 1053

Cattle GGCATGAAGGGGGAGACAGGGTTCCCTGGCCTGCCAGGCTGCAAGGGCTCGCCCGGATTC 1053

Horse GGCATGAAGGGGGAGACAGGGTACCCCGGCCTGCCAGGCTGCAAGGGCTCGCCCGGACTG 1053

Human GGCGTGAAGGGGGAGATGGGGTACCCAGGCCTGCCAGGCTGCAAGGGCTCGCCCGGGTTT 1053

Chimpanzee GGCGTGAAGGGGGAGATGGGGTACCCAGGCCTGCCAGGCTGCAAGGGCTCGCCCGGGTTT 1053

Mouse GGCATGAAGGGAGAGACGGGGTACCCAGGACTACCGGGCTGCAAGGGCTCCCCAGGATTT 1050

Rat GGCATGAAGGGAGAGACGGGATACCCAGGACTACCAGGCTGCAAGGGTTCGCCAGGATTT 1077

*** ******* **** ** * *** ** ** ** *********** ** ** ** *

Buffalo GATGGTATCCAAGGACCCCCCGGGCCCAAGGGTGATGCAGGTGCCTTCGGACTGAAAGGA 1113

Cattle GATGGTATCCAAGGACCCCCCGGGCCCAAGGGTGACCCAGGTGCCTTCGGACTGAAAGGA 1113

Horse GACGGCGTTCAAGGACCTCCCGGGCCCAAGGGCGACGCTGGTGCGTTTGGACTGAAAGGA 1113

Human GACGGCATTCAAGGACCCCCTGGCCCCAAGGGAGACCCCGGCGCCTTTGGACTGAAAGGA 1113

Chimpanzee GACGGCATTCAAGGACCCCCCGGCCCCAAGGGAGACCCCGGCGCCTTTGGACTGAAAGGA 1113

Mouse GATGGCATTCAAGGACCCCCGGGTCCCAAGGGTGATGCTGGTGCCTTTGGGATGAAGGGA 1110

Rat GATGGCATTCAAGGACCCCCAGGTCCTAAGGGCGATGCTGGTGCCTTTGGGCTGAAAGGA 1137

** ** * ******** ** ** ** ***** ** * ** ** ** ** **** ***

Buffalo CAGAAGGGTGAGCCCGGAGCAGACGGGGAGCCTGGGAGGCCAGGGAACACGGGGCCCCCC 1173

Cattle CAGAAGGGTGAGCCCGGAGCAGACGGGGAGCCTGGGAGGCCCGGGAGCACGGGGCCGCCT 1173

Horse GCAAAGGGTGAACCGGGAGCCGATGGAGAGCCCGGGAGGCCGGGGGACACAGGGCCCCCT 1173

Human GAAAAGGGCGAGCCTGGAGCTGACGGGGAGGCGGGGAGACCAGGGAGCTCGGGACCATCT 1173

Chimpanzee GGAAAGGGCGAGCCTGGAGCTGACGGGGAGGCGGGGAGACCAGGGAGCTCGGGACCACCT 1173

Mouse GAAAAGGGTGAAGCTGGAGCAGACGGTGAGGCTGGGAGACCAGGGAACTCAGGGTCACCT 1170

Rat GAAAAGGGTGAAGCTGGAGCAGAAGGTGAGGCTGGGAGACCTGGGAACTCAGGGCCACCC 1197

***** ** * ***** ** ** *** * ***** ** *** * * ** * *

Buffalo GGAGATGAGGGTGAGCCTGGAGAGCCCGGTCCCCCGGGAGAGAAGGGAGAAGCCGGCGAT 1233

Cattle GGAGATGAGGGTGAGCCTGGAGAGCCCGGTCCCCCGGGAGAGAAGGGAGAAGCCGGCGAT 1233

Horse GGAGATGAGGGCGAGCCCGGTGAGCCTGGTCCCCCTGGAGAGAAGGGAGAAGCTGGCGAC 1233

Human GGAGACGAGGGCCAGCCGGGAGAGCCTGGGCCCCCCGGAGAGAAAGGAGAGGCGGGCGAC 1233

Chimpanzee GGAGACGAGGGCCAGCCGGGAGAGCCTGGGCCCCCCGGAGAGAAAGGAGAGGCGGGCGAC 1233

Mouse GGAGATGAGGGTGATCCTGGAGAGCCTGGTCCCCCCGGAGAAAAAGGAGAGGCCGGTGAT 1230

Rat GGAGATGAGGGTGAACCGGGAGAACCTGGTCCCCCCGGAGAAAAAGGAGAGGCCGGTGAT 1257

***** ***** * ** ** ** ** ** ***** ***** ** ***** ** ** **

Buffalo GAGGGAAATGCAGGACCAGACGGAGCCCCCGGAGAGAGGGGCGGCCCTGGGGAAAGAGGA 1293

Cattle GAGGGAAATGCAGGACCAGACGGAGCCCCCGGAGAGAGGGGCGGCCCTGGGGAAAGAGGA 1293

Horse GAGGGAAACTCAGGGCCTGACGGTCCCCCCGGAGACAGGGGCGGCCCTGGGGAAAGGGGA 1293

Human GAGGGGAACCCAGGACCTGACGGTGCCCCCGGGGAGCGGGGTGGCCCTGGAGAGAGAGGA 1293

Chimpanzee GAGGGGAACCCAGGACCTGACGGTGCCCCCGGGGAGCGGGGCGGCCCTGGAGAGAGAGGA 1293

Mouse GAAGGAAATGCTGGCCCAGACGGTGCCCCTGGAGAGAGGGGTGGCCCTGGTGAAAGAGGA 1290

Rat GAGGGAAATGCTGGCCCAGATGGTGCCCCTGGAGAGAGGGGCGGCCCTGGTGAAAGAGGA 1317

** ** ** * ** ** ** ** **** ** ** **** ******** ** ** ***

Buffalo CCTCGAGGGACCCCAGGTGCACGGGGCCCAAGAGGAGACCCGGGTGAAGCTGGACCCCAA 1353

Cattle CCTCGAGGGACCCCAGGTGCACGGGGCCCTAGAGGAGACCCGGGTGAAGCTGGACCCCAA 1353

Horse CCACGGGGGACGCCAGGCGTGCGGGGCCCTAGAGGAGACCCGGGCGAAGCTGGACCCCAA 1353

Human CCACGGGGGACCCCAGGCACACGGGGACCAAGAGGAGACCCTGGTGAAGCTGGCCCGCAG 1353

Chimpanzee CCACGGGGGACCCCAGGCACGCGGGGACCAAGAGGAGACCCTGGTGAAGCTGGCCTGCAG 1353

Mouse CCTCGGGGGACCCCTGGTGTGAGAGGACCAAGGGGAGACCCGGGTGAAGCTGGACCACAG 1350

Rat CCTCGGGGGACCCCTGGTGTGAGGGGCCCAAGGGGAGACCCGGGTGAAGCTGGACCACAA 1377

** ** ***** ** ** * ** ** ** ******** ** ******** * **

Buffalo GGTGACCAGGGACGAGAAGGCCCCGTCGGTGTCCCCGGTGACCCGGGCGAGGCTGGCCCC 1413

Cattle GGTGACCAGGGACGAGAAGGCCCCGTCGGTGTCCCCGGTGACCCGGGCGAGGCTGGCCCC 1413

Horse GGTGACCAGGGACGAGAAGGCCCTGTCGGCGTCCCCGGAGACCCGGGTGAGGCTGGCCCC 1413

Human GGTGATCAGGGAAGAGAAGGCCCCGTTGGTGTCCCTGGAGACCCGGGCGAGGCTGGCCCT 1413

Chimpanzee GGTGATCAGGGAAGAGAAGGCCCCGTTGGTGTCCCGGGAGACCCGGGCGAGGCTGGCCCT 1413

Mouse GGTGACCAAGGAAGAGAGGGGCCCGTCGGCATCCCTGGAGACTCGGGTGAGGCTGGCCCC 1410

Rat GGTGACCAAGGAAGAGAGGGGCCTGTTGGCATCCCTGGAGATCCGGGTGAGAGTGGCCCC 1437

***** ** *** **** ** ** ** ** **** ** ** **** *** ******

Buffalo ATTGGGCCAAAAGGATACCGAGGCGATGAGGGACCCCCTGGGACCGAGGGCCCTAAAGGA 1473

Cattle ATTGGGCCGAAAGGATACCGAGGTGATGAGGGACCCCCTGGGACCGAGGGCCCCAAAGGA 1473

Horse ATCGGACCTAAAGGATACCGAGGAGATGAGGGCCCTCCGGGGCTGGAGGGCCCCAGAGGA 1473

Human ATCGGACCTAAAGGCTACCGAGGCGATGAGGGTCCCCCAGGGTCCGAGGGTGCCAGAGGA 1473

Chimpanzee ATCGGACCTAAAGGCTACCGAGGCGATGAGGGTCCCCCAGGGTCCGAGGGTGCCAGAGGA 1473

Mouse ATTGGACCTAAAGGATACCGAGGTGATGAGGGTCCTCCAGGTCCTGAGGGCCTCAGAGGA 1470

Rat ATCGGACCTAAAGGATACCGAGGTGATGAGGGTCCTCCAGGCCCTGAGGGTCTCAGAGGA 1497

** ** ** ***** ******** ******** ** ** ** ***** * ****

Buffalo GCTCCGGGGCCTGCAGGCCCCCCTGGAGACCCCGGGCTGATGGGTGAGAGGGGTGAAGAC 1533

Cattle GCTCCGGGGCCTGCAGGCCCCCCCGGAGACCCCGGGCTGATGGGTGAGAGGGGTGAAGAC 1533

Horse GCCCCAGGGCCTGCAGGACCCCCCGGAGACCCCGGGCTCATGGGCGCAAGGGGAGAAGAC 1533

Human GCCCCAGGACCTGCCGGACCCCCTGGAGACCCGGGGCTGATGGGTGAAAGGGGAGAAGAC 1533

Chimpanzee GCCCCAGGACCTGCCGGACCCCCTGGAGACCCCGGGCTGATGGGTGAAAGGGGAGAAGAC 1533

Mouse GCCCCAGGACCTGTTGGTCCTCCTGGAGACCCCGGACTGATGGGTGAGAGAGGTGAGGAT 1530

Rat GCCCCAGGACCCGTTGGACCCCCCGGGGACCCAGGACTGATGGGTGAAAGGGGTGAAGAT 1557

** ** ** ** * ** ** ** ** ***** ** ** ***** * ** ** ** **

Buffalo GGCCCCCCCGGGAATGGCACCGAAGGCTTCCCCGGCTTTCCCGGCTATCCAGGCAGCAGA 1593

Cattle GGCCCCCCCGGGAATGGCACTGAAGGCTTCCCCGGCTTTCCCGGCTATCCAGGCAGCAGA 1593

Horse GGCCCCCCCGGAAACGGCACCGAGGGCTTCCCCGGCTTCCCGGGCTATCCAGGCAGCAGG 1593

Human GGCCCCGCTGGAAATGGCACCGAGGGCTTCCCCGGCTTCCCCGGGTATCCGGGCAACAGG 1593

Chimpanzee GGCCCCGCTGGAAATGGCACCGAGGGCTTCCCCGGCTTCCCCGGGTATCCGGGCAACAGG 1593

Mouse GGACCACCAGGAAACGGCACGGAAGGTTTCCCCGGCTTCCCTGGGTATCCAGGCAACAGA 1590

Rat GGACCACCAGGAAATGGCACGGAAGGCTTCCCCGGCTTCCCTGGGTATCCAGGCAACAGA 1617

** ** * ** ** ***** ** ** *********** ** ** ***** **** ***

Buffalo GGTCCTCCTGGGATAAACGGCACCAAAGGCTACCCTGGCCTCAAGGGGGACGAGGGAGAA 1653

Cattle GGTCCTCCTGGGATAAACGGCACCAAAGGCTACCCTGGCCTCAAGGGGGACGAGGGAGAA 1653

Horse GGCCCTCCTGGGATAAACGGCACTAAAGGCTATCCCGGCCTCAAGGGAGACGAAGGAGAA 1653

Human GGCGCTCCCGGGATAAACGGCACGAAGGGCTACCCCGGCCTCAAGGGGGACGAGGGAGAA 1653

Chimpanzee GGCCCTCCCGGGATAAACGGCACGAAGGGCTACCCCGGCCTCAAGGGGGACGAGGGAGAA 1653

Mouse GGCCCTCCTGGGCTAAATGGCACAAAAGGCTACCCTGGCCTCAAGGGGGATGAGGGTGAA 1650

Rat GGCCCTCCTGGGATAAATGGCACGAAGGGCTACCCTGGCCTCAAGGGGGATGAGGGTGAA 1677

** **** *** **** ***** ** ***** ** *********** ** ** ** ***

Buffalo GCCGGGGACCCCGGAGAGGACAATAATGACATTGCTCCACGAGGCGCCAAAGGAGCAAAG 1713

Cattle GCCGGGGACCCCGGAGAGGACAATAATGACATTGCTGCACGAGGCGCCAAAGGAGCAAAG 1713

Horse GCTGGAGACCCCGGAGAGGATAACACTGATATTTCACCCCGTGGTGTCAAAGGAGCGAAG 1713

Human GCCGGGGACCCCGGAGACGATAACAACGACATTGCACCCCGAGGAGTCAAAGGAGCAAAG 1713

Chimpanzee GCCGGGGACCCCGGAGACGATAACAACGACATTGCACCCCAAGGAGTCAAAGGAGCAAAG 1713

Mouse GTGGGAGACCCAGGAGAGGATAACAACGACATTTCACCCCGTGGGGTCAAAGGGGCAAAG 1710

Rat GCCGGAGACCCAGGAGAGGATAACAATGATGTTTCACCCCGTGGAGTCAAAGGGGCAAAG 1737

* ** ***** ***** ** ** * ** ** * * * ** * ****** ** ***

Buffalo GGCTACCGAGGTCCTGAAGGCCCCCAGGGACCCCCAGGACACGTGGGACCACCAGGGCCA 1773

Cattle GGCTACCGAGGTCCTGAAGGCCCCCAGGGACCCCCAGGACACGTGGGACCACCGGGGCCA 1773

Horse GGGTATCGGGGCCCCGAAGGCCCTCCGGGACCCCCAGGACCCCCAGGACGACCAGAGATG 1773

Human GGGTACCGGGGTCCCGAGGGCCCCCAGGGACCCCCAGGACACCAAGGACCGCCTGGGCCG 1773

Chimpanzee GGGTACCGGGGTCCCGAGGGCCCCCAGGGACCCCCAGGACACCAAGGACCGCCTGGGCCG 1773

Mouse GGATACCGAGGCCCAGAAGGACCCCAGGGACCTCCAGGACATGTGGGACCACCTGGGCCA 1770

Rat GGATACCGGGGCCCCGAGGGACCCCAGGGGCCTCCAGGACACGTGGGACCACCTGGGCCA 1797

** ** ** ** ** ** ** ** * *** ** ******* **** ** * *

Buffalo GACGAATGCGAGATTTTGGACATCATCATGAAAATGTGCTCTTGCTGTGAGTGCAAGTGC 1833

Cattle GACGAATGCGAGATTTTGGACATCATCATGAAAATGTGCTCTTGCTGTGAGTGCAAGTGC 1833

Horse GACGAGTGCGAGATTCTGGACATCATCATGAAAATGTGCTCTTGCTGTGAGTGCAAGTGC 1833

Human GACGAATGCGAGATTTTGGACATCATCATGAAAATGTGCTCTTGCTGTGAATGCAAGTGC 1833

Chimpanzee GACGAATGCGAGATTTTGGACATCATCATGAAAATGTGCTCTTGCTGTGAATGCAAGTGC 1833

Mouse GATGAGTGTGAGATCCTGGATATCATCATGAAAATGTGCTCCTGCTGTGAGTGCACATGT 1830

Rat GATGAGTGTGAGATTCTGGATATCATCATGAAAATGTGCTCCTGCTGTGAGTGCACGTGT 1857

** ** ** ***** **** ******************** ******** **** **

Buffalo GGGCCCATCGACATCCTCTTCGTGCTGGACAGCTCCGAGAGCATCGGCCTACAGAACTTC 1893

Cattle GGGCCCATCGACATCCTCTTCGTGCTGGACAGCTCTGAGAGCATCGGCCTACAGAACTTC 1893

Horse GGCCCCATCGACATCCTCTTCGTGTTGGACAGCTCGGAGAGCATCGGCCTGCAGAACTTT 1893

Human GGCCCCATCGACCTCCTGTTCGTGCTGGACAGCTCAGAGAGCATTGGCCTGCAGAACTTC 1893

Chimpanzee GGCCCCATCGACCTCCTGTTCGTGCTGGACAGCTCAGAGAGCATTGGCCTGCAGAACTTC 1893

Mouse GGACCCATTGACATCCTCTTCGTGCTGGACAGCTCGGAGAGCATTGGCCTACAGAACTTT 1890

Rat GGTCCCATCGATATTCTCTTCGTGCTGGATAGCTCAGAGAGCATTGGCCTACAGAACTTT 1917

** ***** ** * ** ****** **** ***** ******** ***** ********

Buffalo GAGATCGCCAAGGACTTCATTGTCAAGGTCATTGACCGGCTGAGCAAGGACGAGCTGGTC 1953

Cattle GAGATCGCCAAGGACTTCATCGTCAAGGTCATTGACCGGCTGAGCAAGGATGAGCTGGTC 1953

Horse GAGATTGCCAAGGACTTCATCGTCAAGGTCATCGACCGGCTGAGCAGGGATGAGCTGGTC 1953

Human GAGATTGCCAAGGACTTCGTCGTCAAGGTCATCGACCGGCTGAGCCGGGACGAGCTGGTC 1953

Chimpanzee GAGATTGCCAAGGACTTCGTCGTCAAGGTCATCGACCGGCTGAGCCGGGACGAGCTGGTC 1953

Mouse GAGATTGCCAAGGACTTCATCATCAAGGTCATTGACCGGTTGAGCAAGGATGAGCTGGTC 1950

Rat GAGATTGCCAAGGACTTCATCATCAAGGTCATTGACCGGCTGAGCAAGGACGAGCTAGTC 1977

***** ************ * ********** ****** ***** *** ***** ***

Buffalo AAGTTTGAGCCTGGGCAGTCGCATGCGGGCGTGGTGCAGTACAGCCACAACCAGATGCAG 2013

Cattle AAGTTTGAGCCTGGGCAGTCGCATGCAGGCGTGGTGCAGTACAGCCACAACCAGATGCAG 2013

Horse AAGTTTGAGGCCGGGCAGTCGCATGCGGGCGTGGTGCAGTACAGCCACAACCAGATGCAG 2013

Human AAGTTCGAGCCAGGGCAGTCGTACGCGGGTGTGGTGCAGTACAGCCACAGCCAGATGCAG 2013

Chimpanzee AAGTTCGAGCCAGGGCAGTCATACGCGGGTGTGGTGCAGTACAGCCACAGCCAGATGCAG 2013

Mouse AAATTTGAGCCAGGGCAGTCTCACGCGGGCGTGGTACAGTACAGCCACAACCAGATGCAA 2010

Rat AAATTTGAGCCAGGGCAGTCCCACGCAGGCGTGGTGCAGTACAGCCACAACCAGATGCAA 2037

** ** *** * ******** * ** ** ***** ************* *********

Buffalo GAGCACGTGGACCTGAGGGACCCCAACATCAGGAGTGCCCAGGACCTCAAGGAGGCCATC 2073

Cattle GAGCACGTGGACCTGAGGGACCCCAACATCAGGAATGCCCAGGACCTCAAGGAGGCCATC 2073

Horse GAGCACGTGGGCCTGAGCGACCCCAACATCAGGAATACCCAGGAGCTCAAGGAAGCCATC 2073

Human GAGCACGTGAGCCTGCGCAGCCCCAGCATCCGGAACGTGCAGGAGCTCAAGGAAGCCATC 2073

Chimpanzee GAGCACGTGAGCCTGCGCAGCCCCAGCATCCGGAACGTGCAGGAGCTCAAGGAAGCCATC 2073

Mouse GAGCACGTGGACATGCGGAGCCCCAACGTCCGCAACGCCCAGGACTTCAAAGAAGCTGTC 2070

Rat GAACACGTGGACATGCGGAGCCCCAACGTCCGCAACGCCCAGGACTTCAAAGAAGCCGTC 2097

** ****** * ** * ***** * ** * * ***** **** ** ** **

Buffalo AAGAAGCTGCAGTGGATGGGCGGCGGCACCTTCACGGGCGAGGCCCTGCAGTACACCCGG 2133

Cattle AAGAAGCTGCAGTGGATGGGCGGCGGCACCTTCACGGGCGAGGCCCTGCAGTACACCCGG 2133

Horse AAGAAGCTGCAGTGGATGGCGGGGGGCACCTTCACGGGAGAGGCTCTGCAGTACACCCGG 2133

Human AAGAGCCTGCAGTGGATGGCGGGCGGCACCTTCACGGGGGAGGCCCTGCAGTACACGCGG 2133

Chimpanzee AAGAGCCTGCAGTGGATGGCGGGCGGCACCTTCACGGGGGAGGCCCTGCAGTACACGCGG 2133

Mouse AAGAAGCTACAATGGATGGCTGGTGGCACATTCACCGGAGAAGCGCTGCAGTACACCCGG 2130

Rat AAGAAACTGCAGTGGATGGCTGGCGGCACATTCACCGGAGAAGCGCTGCAGTACACCCGG 2157

**** ** ** ******* ** ***** ***** ** ** ** *********** ***

Buffalo AGCCGGCTGCTGCCACCCACCCCGAACAACCGCATTGCCCTGGTCATCACTGACGGCCGC 2193

Cattle AGCCGGCTGCTGCCACCCACTCCGAACAACCGCATTGCCCTGGTCATCACCGATGGCCGC 2193

Horse AGCCGGCTGCTGCCGCCCACCCAGAACACCCGGATCGCCTTGGTCATCACGGACGGCCGC 2193

Human GACCAGCTGCTGCCGCCCAGCCCGAACAACCGCATCGCCCTGGTCATCACTGACGGGCGC 2193

Chimpanzee GACCAGCTGCTGCCGCCCAGCCCGAACAACCGCATCGCCCTGGTCATCACTGACGGGCGC 2193

Mouse GACCGGCTACTCCCACCCACACAGAACAACCGAATTGCCCTGGTCATTACGGATGGACGT 2190

Rat GACCGGCTCCTCCCACCCACGCAGAACAACCGAATTGCTCTGGTCATCACAGACGGACGC 2217

** *** ** ** **** * ***** *** ** ** ******* ** ** ** **

Buffalo TCGGACACCCAGAGGGACACCACCCCACTCAGCGTGCTCTGCGGCCCTGACATCCAGGTG 2253

Cattle TCAGACACCCAGAGGGACACCACCCCACTCAGCGTGCTCTGCGGCCCTGACATCCAGGTG 2253

Horse TCAGACACCCAGAGGGACACCACCCCCCTCAGCGTGCTCTGCGGCCCTGACATCCAGGTG 2253

Human TCAGACACTCAGAGGGACACCACACCGCTCAACGTGCTCTGCAGCCCCGGCATCCAGGTG 2253

Chimpanzee TCAGACACTCAGAGGGACACCACACCGCTCAACGTGCTCTGCAGCCCCGGCATCCAGGTG 2253

Mouse TCTGACACTCAACGGGACACGACACCTCTCAGTGTGCTCTGTGGTGCAGACATTCAGGTA 2250

Rat TCTGACACTCAGCGGGACACTACACCCCTCAGTGTGCTCTGCGGCTCAGACATTCAGGTA 2277

** ***** ** ******* ** ** **** ******** * * * *** *****

Buffalo GTCTCCGTGGGCATCAAGGATGTCTTTGGCTTGGCCGCGGGCTCCGACCAGCTCAACGTC 2313

Cattle GTCTCTGTGGGCATCAAGGATGTCTTTGGCTTGGCTGCGGGCTCCGACCAGCTCAACGTC 2313

Horse GTCTCTGTGGGCATCAAGGACGTGTTTGGCTCCATCGCGGGTTCCGACCAGCTCAATGTC 2313

Human GTCTCCGTGGGCATCAAAGACGTGTTTGACTTCATCCCAGGCTCAGACCAGCTCAATGTC 2313

Chimpanzee GTCTCCGTGGGCATCAAAGACGTGTTTGACTTCGTCCCAGGCTCCGACCAGCTCAATGTC 2313

Mouse GTTTCTGTGGGAATCAAGGATGTGTTTGGCTTTGTGGCGGGCTCCGACCAGCTCAATGTC 2310

Rat GTTTCTGTGGGCATCAAGGATGTGTTTGGCTTTGTCGCGGGCTCCGACCAGCTCAACGTC 2337

** ** ***** ***** ** ** **** ** * ** ** *********** ***

Buffalo ATCTCCTGCCAAGGCCTGGCACC---CCAGGGACGGCCGGGCATCTCACTGGTCAAGGAA 2370

Cattle ATCTCCTGCCAAGGCCTGGCACC---CCAGGGACGGCCGGGCATCTCATTGGTCAAGGAA 2370

Horse ATTTCCTGCCAAGGCCTCGCACC---CCAGGGTCGGCCCGGCATCTCACTAGTCAAGGAG 2370

Human ATTTCTTGCCAAGGCCTGGCACCATCCCAGGGCCGGCCCGGCCTCTCGCTGGTCAAGGAG 2373

Chimpanzee ATTTCTTGCCAAGGCCTGGTACCGTCCCAGGGCCGGCCCGGCCTCTCGCTGGTCAAGGAG 2373

Mouse ATTTCCTGCCAAGGCTTATCGC------AAGGTCGGCCAGGTATCTCCCTGGTGAAGGAG 2364

Rat ATTTCCTGCCAAGGCTTATCAC------AAAGCCGGCCGGGTATCTCCCTGGTGAAGGAG 2391

** ** ********* * * * * ***** ** **** * ** *****

Buffalo AACTATGCCGAGCTTCTGGACGATGGCTTCCTGAAGAATATCACCGCCCAGATCTGCATA 2430

Cattle AACTACGCCGAGCTTCTGGACGATGGCTTCCTGAAGAACATCACTGCCCAGATCTGCATA 2430

Horse AACTACGCAGAACTGCTGGAGGACGCCTTCCTCAAGAACATCACCACACAGATCTGCATA 2430

Human AACTATGCAGAGCTGCTGGAGGATGCCTTCCTGAAGAATGTCACCGCCCAGATCTGCATA 2433

Chimpanzee AACTACGCAGAGCTGCTGGAGGATGCCTTCCTGAAGAATGTCACCGCCCAGATCTGCGTA 2433

Mouse AACTATGCAGAGCTTCTCGATGACGGCTTTCTGAAGAACATAACAGCCCAGATCTGTATA 2424

Rat AACTATGCAGAACTTCTAGATGACGGCTTTTTGAAGAACATAACAGCCCAGATCTGTATA 2451

***** ** ** ** ** ** ** * *** * ***** * ** * ******** **

Buffalo GACAAGAAATGTCCAGATTACACCTGCCCAATCATCTTCTCCTCCCCGGCCGACATCACC 2490

Cattle GACAAGAAATGTCCAGATTACAGCTGCCCAATCACCTTCTCCTCCCCGGCCGACATCACC 2490

Horse GACAAGAAGTGTCCAGATTACACCTGCCCAATCACCTTCTCCTCCCCGGCCGACATCACC 2490

Human GACAAGAAGTGTCCAGATTACACCTGCCCCATCACGTTCTCCTCCCCGGCTGACATCACC 2493

Chimpanzee GACAAGAAGTGTCCAGATTACACCTGCCCCATCACGTTCTCCTCCCCGGCTGACATCACC 2493

Mouse GATAAGAAGTGTCCGGATTATACCTGTCCAATCACATTCTCCTCCCCGGCTGACATCACC 2484

Rat GATAAGAAGTGTCCAGATTATACCTGTCCAATCACATTCTCCTCCCCGACCGACATCACC 2511

** ***** ***** ***** * *** ** **** ************ * *********

Buffalo ATCCTCCTGGACGGCTCCGCCAGCGTGGGCAGCCACAACTTTGACATCACCAAACGCTTT 2550

Cattle ATCCTCCTGGACGGCTCCGCCAGCGTGGGCAGCCACAACTTTGACATCACCAAACGCTTT 2550

Horse ATCCTGCTGGACGGGTCGGCCAGCGTGGGCAGCCACAACTTTGACATCACCAAGCGCTTT 2550

Human ATCCTGCTGGACGGCTCCGCCAGCGTGGGCAGCCACAACTTTGACACCACCAAGCGCTTC 2553

Chimpanzee ATCCTGCTGGACGGCTCCGCCAGCGTGGGCAGCCACAACTTTGACACCACCAAGCGCTTC 2553

Mouse ATCCTGCTAGACAGCTCAGCCAGTGTCGGCAGCCACAACTTCGAAACCACCAAGGTCTTC 2544

Rat ATTCTGCTAGACAGCTCGGCCAGTGTCGGCAGCCACAACTTCGAAACCACCAAGGTCTTC 2571

** ** ** *** * ** ***** ** ************** ** * ****** ***

Buffalo GCCAAGCGGCTGGCAGAGCGCTTCCTGACAGCGAGCCGGACAGACCCGGGCCAGGACGTG 2610

Cattle GCCAAGCGGCTGGCAGAGCGCTTCCTGACAGCGAGCCGGACAGACCCGGGCCAGGACGTG 2610

Horse GCCAAGCGGCTGGCTGAGCGCTTCCTGACAGCACGCCGGAAGAACCCAGCCCACGAGGTG 2610

Human GCCAAGCGCCTGGCCGAGCGCTTCCTCACAGCGGGCAGGACGGACCCCGCCCACGACGTG 2613

Chimpanzee GCCAAGCGCCTGGCCGAGCGCTTCCTCACAGCGGGCAGGACGGACCCCGCCCACGACGTG 2613

Mouse GCCAAGCGCCTAGCTGAGCGATTCCTGTCAGCAGGCAGGGCGGATCCTTCCCAGGATGTG 2604

Rat GCCAAGCGCCTAGCCGAGCGGTTCCTGTCAGCGGGCAGGGAAGATCCCACCCAGGTCGTG 2631

******** ** ** ***** ***** **** ** ** * ** *** * ***

Buffalo CGCGTGGCGGTGGTGCAGTACAGTGGCACGGGGCAGCAGCGGCCGGAGCGCGCGGCCCTG 2670

Cattle CGTGTGGCAGTGGTGCAGTACAGCGGTACGGGGCAGCAGCGGCCGGAGCGCGCGGCCCTG 2670

Horse CGGGTCTCGGTGATGCAGTACAGCGGCACGGGGCAGCAGCAGCCGGAGCGCGCGTCCCTG 2670

Human CGGGTGGCGGTGGTGCAGTACAGCGGCACGGGCCAGCAGCGCCCAGAGCGGGCGTCGCTG 2673

Chimpanzee CGGGTGGCGGTGGTGCAGTACAGCGGCACGGGCCAGCAGCGCCCAGAGCGGGCGTCGCTG 2673

Mouse CGGGTGGCCGTGGTACAGTATAGTGGCCAGGGGCAGCAACAGCCAGGTCGGGCGGCTCTT 2664

Rat CGGGTGGCCGTGGTACAGTATAGTGGTCAAGGCCAGCAACAGCCAGGTCGAGCGTCTCTT 2691

** ** * *** * ***** ** ** ** ***** * ** * ** *** * **

Buffalo CAGTTCCTGCAGAACTACACGGTGCTGGCCAACACCGTGGACTCCATGGACTTCTTCAAT 2730

Cattle CAGTTCCTGCAGAACTACACCGTGCTGGCCAACACCGTGGACTCCATGGACTTCTTCAAT 2730

Horse CAGTTCCTGCAGAACTACACGGTGCTGGCCAGCACCATCGACAGCATGGACTTCATCAAC 2730

Human CAGTTCCTGCAGAACTACACGGCCCTGGCCAGTGCCGTCGATGCCATGGACTTTATCAAC 2733

Chimpanzee CAGTTCCTGCAGAACTACACGGCCCTGGCCAGTGCCGTCGATGCCATGGACTTTATCAAC 2733

Mouse CAGTTCTTACAGAATTACACAGTGCTGGCCAGCTCTGTGGACAGCATGGATTTCATCAAC 2724

Rat CAGTTCCAGCAGAACTACACGGTACTGGCCAGCTCTGTGGACAGCATGGATTTCATCAAC 2751

****** ***** ***** * ******* * * ** ****** ** ****

Buffalo GACGCCACTGACGTCATAGACGCCCTGGGCTACGTGACCCGCTTCTACCGCGAGGCCTCA 2790

Cattle GACGCCACCGACGTCATGGACGCCCTGGGCTACGTGACCCGCTTCTACCGCGAGGCCTCG 2790

Horse GATGCCACCGACGTCACCGATGCCCTCAGCTACGTGACCCGCTTCTACCGCGAGGCCTCG 2790

Human GACGCCACCGACGTCAACGATGCCCTGGGCTATGTGACCCGCTTCTACCGCGAGGCCTCG 2793

Chimpanzee GACGCCACTGACGTCAACGACGCCCTGGGCTATGTGACCCGCTTCTACCGCGAGGCCTCG 2793

Mouse GACGCCACAGACGTCAACGATGCTCTGAGCTACGTGACTCGTTTCTACCGGGAAGCCTCG 2784

Rat GACGCCACAGATGTCAACGACGCTCTGAGCTATGTAACTCGTTTCTACCGGGAAAACTCC 2811

** ***** ** **** ** ** ** **** ** ** ** ******** ** ***

Buffalo TCCACTGCCGCCAAGAAGAGGCTGCTGCTCTTCTCGGATGGCAACTCGCAGGGCGCCACG 2850

Cattle TCCAACGCCGCCAAGAAGAGGCTGCTGCTCTTCTCGGATGGCAACTCGCAAGGTGCCACG 2850

Horse TCGGAGGAGGTCGATAGGAGGCTGCTGCTCTTCTCAGACGGCAACTCGCAGGGGGCCACG 2850

Human TCCGGCGCTGCCAAGAAGAGGCTGCTGCTCTTCTCAGATGGCAACTCGCAGGGCGCCACG 2853

Chimpanzee TCCGGCGCTGCTAAGAAGAGGCTGCTGCTCTTCTCAGATGGCAACTCGCAGGGCGCCACG 2853

Mouse TCAGGTGCCACCAAGAAGAGAGTGCTGTTGTTTTCAGACGGCAACTCTCAGGGGGCCACA 2844

Rat TCAGGTGCCACCAAGAAGAGAGTGCTGTTGTTCTCAGATGGTAACTCACAGGGGGCCACA 2871

** * * * *** ***** * ** ** ** ** ***** ** ** *****

Buffalo CCGGCCACCATCGAGAAGGCGGTACAGGAGGCCCAGCGGGCGGGCCTGGAGATCTTCGCG 2910

Cattle CCGGCCGCCATCGAGAAGGCGGTGCAGGAGGCCCAGCGGGCGGGCGTGGAGATCTTCGCG 2910

Horse GCGGCGGCCATCAAGAAGGCCGTGCAGGAGGCCCAGCGGGCGAACATCGAGGTCTTCGTG 2910

Human CCCGCTGCCATCGAGAAGGCCGTGCAGGAAGCCCAGCGGGCAGGCATCGAGATCTTCGTG 2913

Chimpanzee CCCGCTGCCATCGAGAAGGCCGTGCAGGAAGCCCAGCGGGCAGGCATCGAGATCTTCGTG 2913

Mouse GCAGAGGCCATTGAGAAGGCTGTGCAGGAGGCCCAGCGTGCAGGCATTGAGATCTTTGTG 2904

Rat GCAGAGGCCATTGAGAAGGCTGTGCAGGAGGCCCAGCGTGGGGGCATTGAGATCTTTGTG 2931

* * **** ******* ** ***** ******** * * * *** **** * *

Buffalo GTGGTGGTGGGCCGCCAGGTGAACGAGCCCCACGTGCGTGTCCTGGTCACCGGCAAGGCG 2970

Cattle GTGGTGGTGGGCCGCCAGGTGAACGAGCCCCACGTGCGTGTCCTGGTCACTGGCAAGGCG 2970

Horse GTGGTGGTGGGCACCCACGTGAACGAGCCCCACATTCGCGTCCTGGTCACGGACAAGACG 2970

Human GTGGTCGTGGGCCGCCAGGTGAATGAGCCCCACATCCGCGTCCTGGTCACCGGCAAGACG 2973

Chimpanzee GTGGTCGTGGGCCGCCAGGTGAATGAGCCCCACATCCGCGTCCTGGTCACCGGCAAGACG 2973

Mouse GTGGTGGTGGGACCCCAGGTGAACGAGCCCCACATCCGTGTGCTTGTCACTGGCAAGACT 2964

Rat ATGGTGGTGGGACCCCAGGTGAATGAACCCCACATCCGTGTACTCGTCACCGGTAAGACC 2991

**** ***** *** ***** ** ****** * ** ** ** ***** * *** *

Buffalo GCTGAGTATGACGTGGTCTTCGGCGAGCGCCACCTGTTCCGCGTGCCCAGCTACCAGGCG 3030

Cattle GCTGAGTATGACGTGGTCTTCGGCGAGCGCCACCTGTTCCGCGTGCCCAGTTACCAGGCG 3030

Horse GCCGAGTACAACGTGGCCTATGGCCAGCGCCACCAGTTCCGCGTGGCCAGCTACCAGGAG 3030

Human GCCGAGTACGACGTGGCCTACGGCGAGAGCCACCTGTTCCGTGTCCCCAGCTACCAGGCC 3033

Chimpanzee GCCGAGTACGACGTGGCCTACGGCGAGCGCCACCTGTTCCGTGTCCCCAGCTACCAGGCC 3033

Mouse GCAGAGTACGACGTGGCCTTTGGCGAGCGCCACCTATTCCGTGTACCAAACTACCAGGCC 3024

Rat GCCGAGTACGACGTGGCCTTCGGCGAGCGCCACCTATTCCGTGTACCGAACTACCAGGCC 3051

** ***** ****** ** *** ** ****** ***** ** * * *******

Buffalo TTGCTGCGGGGTGTCTTCTACCAGACGGTGTCCAGGAAGGTGGCGCTGGACTAG 3084

Cattle TTGCTGCGGGGCGTCTTCTACCAGACGGTGTCCAGGAAGGTGGCGCTGGACTAG 3084

Horse CTGCTTGGCGGCGTCTTCTACCAGTCGGTGTCCAGGAAGGTGGCCATGGGCTAG 3084

Human CTGCTCCGCGGTGTCTTCCACCAGACAGTCTCCAGGAAGGTGGCGCTGGGCTAG 3087

Chimpanzee CTGCTCCGCGGTGTCTTCCACCAGACAGTCTCCAGGAAGGTGGCGCTGGGCTAG 3087

Mouse CTGCTACGTGGCGTACTCTACCAGACAGTCTCCAGGAAGGTGGCACTGGGCTAG 3078

Rat CTACTACGTGGTGTCCTCTACCAGACAGTCTCCAGGAAGGTGGCCCTGGGCTAG 3105

* ** * ** ** ** ***** * ** ************** *** ****

**(ii) Phylogenetic tree based on nucleotide sequence**


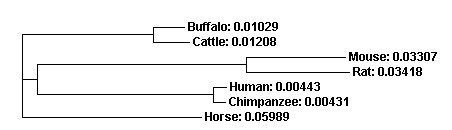


**(iii) Multiple amino acid sequence alignment of COL6A1 protein of different species**

Buffalo ---------MRLPRALLPLLLQACWASAQDDTVASRAIAFQDCPVDLFFVLDTSESVALR 51

Cattle ---------MRLPRALLPLLLQACWASAQDDPVASRAIAFQDCPVDLFFVLDTSESVALR 51

Mouse ---------MRLAHALLPLLLQACWVATQDIQGS-KAIAFQDCPVDLFFVLDTSESVALR 50

Rat MVTRRPAGNMRLAHTLLPLLLQACWVAAQDIQGS-RAIAFQDCPVDLFFVLDTSESVALR 59

Human ---------MRAARALLPLLLQACWTAAQDEPETPRAVAFQDCPVDLFFVLDTSESVALR 51

Chimpanzee ---------MRAARALLPLLLQACWTAAQDEPETPRAVAFQDCPVDLFFVLDTSESVALR 51

Horse ---------MRLASALLLLLLQACWAAAQDDTAAVRTVAFQDCPVDLFFVLDTSESVALR 51

** . :** *******.::** : :::**********************

Buffalo LKPYGALVDKVKSFTKRFIDNLKDRYYRCDRNLVWNAGALHYSDEVEIIRGLTRMPSGRD 111

Cattle LKPYGALVDKVKSFTKRFIDNLNDRYYRCDRNLVWNAGALHYSDEVEIIRGLTRMPSGRD 111

Mouse LKPYGALVDKVKSFTKRFIDNLRDRYYRCDRNLVWNAGALHYSDEVEIIRGLTRMPSGRD 110

Rat LKPYGALVDKVKSFTKRFIDNLRDRYYRCDRNLVWNAGALHYSDEVEIIRGLMRMPSGRD 119

Human LKPYGALVDKVKSFTKRFIDNLRDRYYRCDRNLVWNAGALHYSDEVEIIQGLTRMPGGRD 111

Chimpanzee LKPYGALVDKVKSFTKRFIDNLRDRYYRCDRNLVWNAGALHYSDEVEIIQGLTRMPGGRD 111

Horse LKPYGALVDKVKAFTKRFIDNLRDRYYRCDRNLVWNAGALHYSDEVEIIRGLTRMPSGRD 111

************:*********.**************************:** ***.***

Buffalo ELKSSVDAVKYFGKGTYTDCAIKKGLEELLVGGSHLKENKYLVVVTDGHPLEGYKEPCGG 171

Cattle ELKSSVDAVKYFGKGTYTDCAIKKGLEELLVGGSHLKENKYLVVVTDGHPLEGYKEPCGG 171

Mouse ELKASVDAVKYFGKGTYTDCAIKKGLEELLIGGSHLKENKYLIVVTDGHPLEGYKEPCGG 170

Rat ELKASIDAVKYFGKGTYTDCAIKKGLEELLIGGSHLKENKYLIVVTDGHPLEGYKEPCGG 179

Human ALKSSVDAVKYFGKGTYTDCAIKKGLEQLLVGGSHLKENKYLIVVTDGHPLEGYKEPCGG 171

Chimpanzee ALKSSVDAVKYFGKGTYTDCAIKKGLEQLLVGGSHLKENKYLIVVTDGHPLEGYKEPCGG 171

Horse ELKASVDAVKYFGKGTYTDCAIKKGLEELLVGGSHLKENKYLIVVTDGHPLEGYKEPCGG 171

**:*:*********************:**:***********:*****************

Buffalo LEDAVNEAKHLGIKVFSVAITPDHLEPRLSIIATDHTYRRNFTAADWGQSRDAEEVISQT 231

Cattle LEDAVNEAKHLGIKVFSVAITPDHLEPRLSIIATDHTYRRNFTAADWGQSRDAEEVISQT 231

Mouse LEDAVNEAKHLGIKVFSVAITPDHLEPRLSIIATDHTYRRNFTAADWGHSRDAEEVISQT 230

Rat LEDAVNEAKHLGIKVFSVAITPDHLEPRLSIIATDHTYRRNFTAADWGHSRDAEETISQT 239

Human LEDAVNEAKHLGVKVFSVAITPDHLEPRLSIIATDHTYRRNFTAADWGQSRDAEEAISQT 231

Chimpanzee LEDAVNEAKHLGVKVFSVAITPDHLEPRLSIIATDHTYRRNFTAADWGQSRDAEEAISQT 231

Horse LEDAVNEAKHLGIKVFSVAITPDHLEPRLSIIATDHTYRRNFTAADWGQSRDAEEIISQT 231

************:***********************************:****** ****

Buffalo IETITDMIKNNVEQVCCSFECQPARGPPGPRGDPGYEGERGKPGLPGEKGEAGDPGRPGD 291

Cattle IDTITDMIKNNVEQVCCSFECQPARGPPGPRGDPGYEGERGKPGLPGEKGEAGDPGRPGD 291

Mouse IDTIVDMIKNNVEQVCCSFECQAARGPPGPRGDPGYEGERGKPGLPGEKGEAGDPGRPGD 290

Rat IDTIVDMIKNNVEQVCCTFECQAARGPPGPRGDPGYEGERGKPGLPGEKGEAGDPGRPGD 299

Human IDTIVDMIKNNVEQVCCSFECQPARGPPGLRGDPGFEGERGKPGLPGEKGEAGDPGRPGD 291

Chimpanzee IDTIVDMIKNNVEQVCCSFECQPARGPPGLRGDPGFEGERGKPGLPGEKGEAGDPGRPGD 291

Horse IDTITDMIKNNVEQVCCSFECQPARGPPGLRGDPGYEGERGKPGLPGEKGEAGDPGRPGD 291

*:**.************:****.****** *****:************************

Buffalo LGPVGYQGMKGEKGSRGEKGSRGPKGYKGEKGKRGMDGVDGMKGETGYPGLPGCKGSPGF 351

Cattle LGPVGYQGMKGEKGSRGEKGSRGPKGYKGEKGKRGMDGVDGMKGETGFPGLPGCKGSPGF 351

Mouse LGPVGYQGMKGEKGSRGEKGSRGPKGYKGEKGKRGIDGVDGMKGETGYPGLPGCKGSPGF 350

Rat LGPVGYQGMKGEKGSRGEKGSRGPKGYKGEKGKRGIDGVDGMKGETGYPGLPGCKGSPGF 359

Human LGPVGYQGMKGEKGSRGEKGSRGPKGYKGEKGKRGIDGVDGVKGEMGYPGLPGCKGSPGF 351

Chimpanzee LGPVGYQGMKGEKGSRGEKGSRGPKGYKGEKGKRGIDGVDGVKGEMGYPGLPGCKGSPGF 351

Horse LGPVGYQGMKGEKGSRGDKGSRGPKGYKGEKGKRGIDGVDGMKGETGYPGLPGCKGSPGL 351

*****************:*****************:*****:*** *:***********:

Buffalo DGIQGPPGPKGDAGAFGLKGQKGEPGADGEPGRPGNTGPPGDEGEPGEPGPPGEKGEAGD 411

Cattle DGIQGPPGPKGDPGAFGLKGQKGEPGADGEPGRPGSTGPPGDEGEPGEPGPPGEKGEAGD 411

Mouse DGIQGPPGPKGDAGAFGMKGEKGEAGADGEAGRPGNSGSPGDEGDPGEPGPPGEKGEAGD 410

Rat DGIQGPPGPKGDAGAFGLKGEKGEAGAEGEAGRPGNSGPPGDEGEPGEPGPPGEKGEAGD 419

Human DGIQGPPGPKGDPGAFGLKGEKGEPGADGEAGRPGSSGPSGDEGQPGEPGPPGEKGEAGD 411

Chimpanzee DGIQGPPGPKGDPGAFGLKGGKGEPGADGEAGRPGSSGPPGDEGQPGEPGPPGEKGEAGD 411

Horse DGVQGPPGPKGDAGAFGLKGAKGEPGADGEPGRPGDTGPPGDEGEPGEPGPPGEKGEAGD 411

**:*********.****:** ***.**:**.****.:*..****:***************

Buffalo EGNAGPDGAPGERGGPGERGPRGTPGARGPRGDPGEAGPQGDQGREGPVGVPGDPGEAGP 471

Cattle EGNAGPDGAPGERGGPGERGPRGTPGARGPRGDPGEAGPQGDQGREGPVGVPGDPGEAGP 471

Mouse EGNAGPDGAPGERGGPGERGPRGTPGVRGPRGDPGEAGPQGDQGREGPVGIPGDSGEAGP 470

Rat EGNAGPDGAPGERGGPGERGPRGTPGVRGPRGDPGEAGPQGDQGREGPVGIPGDPGESGP 479

Human EGNPGPDGAPGERGGPGERGPRGTPGTRGPRGDPGEAGPQGDQGREGPVGVPGDPGEAGP 471

Chimpanzee EGNPGPDGAPGERGGPGERGPRGTPGTRGPRGDPGEAGLQGDQGREGPVGVPGDPGEAGP 471

Horse EGNSGPDGPPGDRGGPGERGPRGTPGVRGPRGDPGEAGPQGDQGREGPVGVPGDPGEAGP 471

***.****.**:**************.*********** ***********:***.**:**

Buffalo IGPKGYRGDEGPPGTEGPKGAPGPAGPPGDPGLMGERGEDGPPGNGTEGFPGFPGYPGSR 531

Cattle IGPKGYRGDEGPPGTEGPKGAPGPAGPPGDPGLMGERGEDGPPGNGTEGFPGFPGYPGSR 531

Mouse IGPKGYRGDEGPPGPEGLRGAPGPVGPPGDPGLMGERGEDGPPGNGTEGFPGFPGYPGNR 530

Rat IGPKGYRGDEGPPGPEGLRGAPGPVGPPGDPGLMGERGEDGPPGNGTEGFPGFPGYPGNR 539

Human IGPKGYRGDEGPPGSEGARGAPGPAGPPGDPGLMGERGEDGPAGNGTEGFPGFPGYPGNR 531

Chimpanzee IGPKGYRGDEGPPGSEGARGAPGPAGPPGDPGLMGERGEDGPAGNGTEGFPGFPGYPGNR 531

Horse IGPKGYRGDEGPPGLEGPRGAPGPAGPPGDPGLMGARGEDGPPGNGTEGFPGFPGYPGSR 531

************** ** :*****.********** ******.***************.*

Buffalo GPPGINGTKGYPGLKGDEGEAGDPGEDNNDIAPRGAKGAKGYRGPEGPQGPPGHVGPPGP 591

Cattle GPPGINGTKGYPGLKGDEGEAGDPGEDNNDIAARGAKGAKGYRGPEGPQGPPGHVGPPGP 591

Mouse GPPGLNGTKGYPGLKGDEGEVGDPGEDNNDISPRGVKGAKGYRGPEGPQGPPGHVGPPGP 590

Rat GPPGINGTKGYPGLKGDEGEAGDPGEDNNDVSPRGVKGAKGYRGPEGPQGPPGHVGPPGP 599

Human GAPGINGTKGYPGLKGDEGEAGDPGDDNNDIAPRGVKGAKGYRGPEGPQGPPGHQGPPGP 591

Chimpanzee GPPGINGTKGYPGLKGDEGEAGDPGDDNNDIAPQGVKGAKGYRGPEGPQGPPGHQGPPGP 591

Horse GPPGINGTKGYPGLKGDEGEAGDPGEDNTDISPRGVKGAKGYRGPEGPPGPPGPPGRPEM 591

*.**:***************.****:**.*::.:*.************ **** * *

Buffalo DECEILDIIMKMCSCCECKCGPIDILFVLDSSESIGLQNFEIAKDFIVKVIDRLSKDELV 651

Cattle DECEILDIIMKMCSCCECKCGPIDILFVLDSSESIGLQNFEIAKDFIVKVIDRLSKDELV 651

Mouse DECEILDIIMKMCSCCECTCGPIDILFVLDSSESIGLQNFEIAKDFIIKVIDRLSKDELV 650

Rat DECEILDIIMKMCSCCECTCGPIDILFVLDSSESIGLQNFEIAKDFIIKVIDRLSKDELV 659

Human DECEILDIIMKMCSCCECKCGPIDLLFVLDSSESIGLQNFEIAKDFVVKVIDRLSRDELV 651

Chimpanzee DECEILDIIMKMCSCCECKCGPIDLLFVLDSSESIGLQNFEIAKDFVVKVIDRLSRDELV 651

Horse DECEILDIIMKMCSCCECKCGPIDILFVLDSSESIGLQNFEIAKDFIVKVIDRLSRDELV 651

******************.*****:*********************::*******:****

Buffalo KFEPGQSHAGVVQYSHNQMQEHVDLRDPNIRSAQDLKEAIKKLQWMGGGTFTGEALQYTR 711

Cattle KFEPGQSHAGVVQYSHNQMQEHVDLRDPNIRNAQDLKEAIKKLQWMGGGTFTGEALQYTR 711

Mouse KFEPGQSHAGVVQYSHNQMQEHVDMRSPNVRNAQDFKEAVKKLQWMAGGTFTGEALQYTR 710

Rat KFEPGQSHAGVVQYSHNQMQEHVDMRSPNVRNAQDFKEAVKKLQWMAGGTFTGEALQYTR 719

Human KFEPGQSYAGVVQYSHSQMQEHVSLRSPSIRNVQELKEAIKSLQWMAGGTFTGEALQYTR 711

Chimpanzee KFEPGQSYAGVVQYSHSQMQEHVSLRSPSIRNVQELKEAIKSLQWMAGGTFTGEALQYTR 711

Horse KFEAGQSHAGVVQYSHNQMQEHVGLSDPNIRNTQELKEAIKKLQWMAGGTFTGEALQYTR 711

***.***:********.******.: .*.:*..*::***:*.****.*************

Buffalo SRLLPPTPNNRIALVITDGRSDTQRDTTPLSVLCGPDIQVVSVGIKDVFGLAAGSDQLNV 771

Cattle SRLLPPTPNNRIALVITDGRSDTQRDTTPLSVLCGPDIQVVSVGIKDVFGLAAGSDQLNV 771

Mouse DRLLPPTQNNRIALVITDGRSDTQRDTTPLSVLCGADIQVVSVGIKDVFGFVAGSDQLNV 770

Rat DRLLPPTQNNRIALVITDGRSDTQRDTTPLSVLCGSDIQVVSVGIKDVFGFVAGSDQLNV 779

Human DQLLPPSPNNRIALVITDGRSDTQRDTTPLNVLCSPGIQVVSVGIKDVFDFIPGSDQLNV 771

Chimpanzee DQLLPPSPNNRIALVITDGRSDTQRDTTPLNVLCSPGIQVVSVGIKDVFDFVPGSDQLNV 771

Horse SRLLPPTQNTRIALVITDGRSDTQRDTTPLSVLCGPDIQVVSVGIKDVFGSIAGSDQLNV 771

.:****: *.********************.***...************. .*******

Buffalo ISCQGLAP-QGRPGISLVKENYAELLDDGFLKNITAQICIDKKCPDYTCPIIFSSPADIT 830

Cattle ISCQGLAP-QGRPGISLVKENYAELLDDGFLKNITAQICIDKKCPDYSCPITFSSPADIT 830

Mouse ISCQGLS--QGRPGISLVKENYAELLDDGFLKNITAQICIDKKCPDYTCPITFSSPADIT 828

Rat ISCQGLS--QSRPGISLVKENYAELLDDGFLKNITAQICIDKKCPDYTCPITFSSPTDIT 837

Human ISCQGLAPSQGRPGLSLVKENYAELLEDAFLKNVTAQICIDKKCPDYTCPITFSSPADIT 831

Chimpanzee ISCQGLVPSQGRPGLSLVKENYAELLEDAFLKNVTAQICVDKKCPDYTCPITFSSPADIT 831

Horse ISCQGLAP-QGRPGISLVKENYAELLEDAFLKNITTQICIDKKCPDYTCPITFSSPADIT 830

****** *.***:***********:*.****:*:***:*******:*** ****:***

Buffalo ILLDGSASVGSHNFDITKRFAKRLAERFLTASRTDPGQDVRVAVVQYSGTGQQRPERAAL 890

Cattle ILLDGSASVGSHNFDITKRFAKRLAERFLTASRTDPGQDVRVAVVQYSGTGQQRPERAAL 890

Mouse ILLDSSASVGSHNFETTKVFAKRLAERFLSAGRADPSQDVRVAVVQYSGQGQQQPGRAAL 888

Rat ILLDSSASVGSHNFETTKVFAKRLAERFLSAGREDPTQVVRVAVVQYSGQGQQQPGRASL 897

Human ILLDGSASVGSHNFDTTKRFAKRLAERFLTAGRTDPAHDVRVAVVQYSGTGQQRPERASL 891

Chimpanzee ILLDGSASVGSHNFDTTKRFAKRLAERFLTAGRTDPAHDVRVAVVQYSGTGQQRPERASL 891

Horse ILLDGSASVGSHNFDITKRFAKRLAERFLTARRKNPAHEVRVSVMQYSGTGQQQPERASL 890

****.*********: ** **********:* * :* : ***:*:**** ***:* **:*

Buffalo QFLQNYTVLANTVDSMDFFNDATDVIDALGYVTRFYREASSTAAKKRLLLFSDGNSQGAT 950

Cattle QFLQNYTVLANTVDSMDFFNDATDVMDALGYVTRFYREASSNAAKKRLLLFSDGNSQGAT 950

Mouse QFLQNYTVLASSVDSMDFINDATDVNDALSYVTRFYREASSGATKKRVLLFSDGNSQGAT 948

Rat QFQQNYTVLASSVDSMDFINDATDVNDALSYVTRFYRENSSGATKKRVLLFSDGNSQGAT 957

Human QFLQNYTALASAVDAMDFINDATDVNDALGYVTRFYREASSGAAKKRLLLFSDGNSQGAT 951

Chimpanzee QFLQNYTALASAVDAMDFINDATDVNDALGYVTRFYREASSGAAKKRLLLFSDGNSQGAT 951

Horse QFLQNYTVLASTIDSMDFINDATDVTDALSYVTRFYREASSEEVDRRLLLFSDGNSQGAT 950

** ****.**.::*:***:****** ***.******** ** ..:*:************

Buffalo PATIEKAVQEAQRAGLEIFAVVVGRQVNEPHVRVLVTGKAAEYDVVFGERHLFRVPSYQA 1010

Cattle PAAIEKAVQEAQRAGVEIFAVVVGRQVNEPHVRVLVTGKAAEYDVVFGERHLFRVPSYQA 1010

Mouse AEAIEKAVQEAQRAGIEIFVVVVGPQVNEPHIRVLVTGKTAEYDVAFGERHLFRVPNYQA 1008

Rat AEAIEKAVQEAQRGGIEIFVMVVGPQVNEPHIRVLVTGKTAEYDVAFGERHLFRVPNYQA 1017

Human PAAIEKAVQEAQRAGIEIFVVVVGRQVNEPHIRVLVTGKTAEYDVAYGESHLFRVPSYQA 1011

Chimpanzee PAAIEKAVQEAQRAGIEIFVVVVGRQVNEPHIRVLVTGKTAEYDVAYGERHLFRVPSYQA 1011

Horse AAAIKKAVQEAQRANIEVFVVVVGTHVNEPHIRVLVTDKTAEYNVAYGQRHQFRVASYQE 1010

. :*:********..:*:*.:*** :*****:*****.*:***:*.:*: * ***..**

Buffalo LLRGVFYQTVSRKVALD 1027

Cattle LLRGVFYQTVSRKVALD 1027

Mouse LLRGVLYQTVSRKVALG 1025

Rat LLRGVLYQTVSRKVALG 1034

Human LLRGVFHQTVSRKVALG 1028

Chimpanzee LLRGVFHQTVSRKVALG 1028

Horse LLGGVFYQSVSRKVAMG 1027

** **::*:******:.

**(iv) Phylogenetic tree based on amino acid sequence**


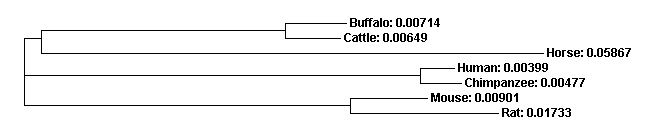

Supplement: Figure S2 — Multiple alignment of the COL6A1 gene (i), phylogenetic tree based on nucleotide sequence (ii), multiple alignment of amino acid sequence of the COL6A1 protein (iii) and phylogenetic tree based on amino acid sequence (iv) of different species. Note the close relationship between cattle and water buffalo in the phylogenetic tree. Horse, as expected, has a distant relationship with water buffalo and cattle, whereas mouse and rat group together. (DOC) [file pone.0024958.s002.doc]
