# Supplementary material for: Molecular Mining of Alleles in Water Buffalo Bubalus bubalis and Characterization of the TSPY1 and COL6A1 Genes
Source: PLoS One. 2011 Sep 15;6(9):e24958. doi: 10.1371/journal.pone.0024958 (PMC3174239; doi:10.1371/journal.pone.0024958)
Supplement: Table S1 — List of primers used to test for genomic DNA contamination in the samples. The primers for ACTB were designed in our lab, while those for CD45 and CDH1 genes were based on an earlier report [31]. Primers corresponding to CD45 and CDH1 span several introns but their positions were not defined. (DOC) [file pone.0024958.s003.doc]

**Table S1**: List of the primers used for the verification of genomic DNA contamination in the sample

| **Gene** | **Accession no.** | **Primer sequence 5’-3’** | **Product Size(in bp)**  **cDNA** | **Product Size(in bp)**  **gDNA** |
| --- | --- | --- | --- | --- |
| ACTB | DQ661647 | F CAGATCATGTTCGAGACCTTCAA  R GATGATCTTGATCTTCATTGTGCTG | 630 | 720 |
| PRM1 | NM_174156 | F AGATACCGATGCTGCCTCAC  R GTGGCATGTTCAAGATGTGG | 234 | 334 |
| CD45 | AJ400864 | F GACATCGCAGTGTTTGTTGC  R GGAGGTTCACATTCCTCTCG | 229 | Undefined |
| CDH1 | NM_001002763 | F TCTACAGCATCACTGGCCAACGAGCTG  R TGCTTGGACCATCAGGGTGTATGTGGG | 476 | Undefined |
