# Supplementary material for: Molecular Mining of Alleles in Water Buffalo Bubalus bubalis and Characterization of the TSPY1 and COL6A1 Genes
Source: PLoS One. 2011 Sep 15;6(9):e24958. doi: 10.1371/journal.pone.0024958 (PMC3174239; doi:10.1371/journal.pone.0024958)
Supplement: Table S2 — Detailed analysis of MASA identified somatic, gonadal and spermatozoal mRNA transcripts tagged with the TGG repeat from the water buffalo Bubalus bubalis. (i), Transcripts identified from the somatic and gonadal tissues. (ii), Transcripts identified from spermatozoa. All transcripts, their accession number and their homology status are listed in this table. (DOC) [file pone.0024958.s004.doc]

**Table S2: Details of the MASA identified somatic, gonadal and spermatozoal mRNA transcripts tagged with TGG repeat motif from water buffalo *Bubalus bubalis*.**

1. mRNA transcripts uncovered from different tissues

| Transcript ID | Clone ID | Accession no. | Size(in bp) | Tissue Origin | Details of homology status | Accession no of similar genes | Gene length(bp) | Chromosomal location | Position of (TGG)_5_ tagged transcript | Score | QC% | E-value | Similarity % |
| --- | --- | --- | --- | --- | --- | --- | --- | --- | --- | --- | --- | --- | --- |
| SR 1 | pSRC1  pSRC2  pSRC3  pSRC4  pSRC5  pSRC6  pSRC7 | GU433047  GU433048  GU433049  GU433050  GU433051  GU433052  GU433053 | 507  507  507  510  507  522  510 | Heart  Kidney  Liver  Lung  Spleen  Testis  Ovary | 1) Bos taurus collagen, type VI, alpha 1 (COL6A1), mRNA  2) PREDICTED: Canis familiaris collagen VI-alpha1 protein (COL6A1), | NM_001143865.1  XM_548729.2 | mRNA =4069  mRNA =621 | 1q12-q14  31 | 3129-3626  445-621 | 824  272 | 97%  34% | 0.0  1e-69 | 96%  94% |
| SR2 | pSRC8  pSRC9  pSRC10  pSRC11  pSRC12 | GU433054  GU433055  GU433056  GU433057  GU433058 | 465  465  465  465  465 | Heart  Liver  Lung  Testis  Ovary | 1) Bos taurus alpha-2-HS-glycoprotein (AHSG), mRNA >emb|X16577.1| B.taurus mRNA for fetuin  2) Ovis aries fetuin (LOC443392), mRNA >emb|X16578.1| Ovis aries mRNA for fetuin | NM_173984.2  NM_001009802.1 | mRNA =1459  mRNA =1518 | 1  - | 882-1337  941-1389 | 813  691 | 97%  97% | 0.0  0.0 | 98%  94% |
| SR3 | pSRC13  pSRC14 | GU433059  GU433060 | 495  495 | Heart  Liver | 1) Homo sapiens TEK tyrosine kinase, endothelial (TEK) on chromosome 9  2) Homo sapiens chromosome 9 BAC RP11-179D22, complete sequence | NG_011828.1  AL355433.6 | Gene length =128026  Gene length =177219 | 9p21  9 | 113007-113160  6102-5949 | 77.0  77.0 | 30%  30% | 9e-11  9e-11 | 71%  71% |
| SR4 | pSRC15 | GU433061 | 498 | Heart | 1) Homo sapiens BAC clone RP11-521D12 from 2, complete sequence | AC082651.6 | Gene Length =203770 | 2 | 24668-24643 | 49.1 | 5% | 0.022 | 100% |
| SR5 | pSRC16 | GU433062 | 471 | Heart | 1) PREDICTED: Sus scrofa similar to sorbin and SH3 domain containing 1 (LOC100153151), mRNA  2) Rattus norvegicus 1 BAC CH230-145B9 (Children's Hospital Oakland Research Institute) complete sequence | XM_001924661.1  AC109039.10 | mRNA =4160  Gene length =215780 | 14  1 | 3267-3281  32071-31912 | 169  163 | 24%  33% | 2e-38  7e-37 | 93%  85% |
| SR6 | pSRC17 | GU433063 | 394 | Heart | 1) Bos taurus family with sequence similarity 50, member A (FAM50A), mRNA  2) PREDICTED: Equus caballus misc_RNA (LOC100058349), miscRNA  3) Homo sapiens family with sequence similarity 50, member A (FAM50A), mRNA | NM_001034496.2  XR_044493.1  NM_004699.2 | mRNA =1517  mRNA =6857  mRNA =1357 | X  X  X | 482-861  430-808  509-884 | 675  534  507 | 96%  96%  95% | 0.0  1e-148  2e-140 | 98%  92%  91% |
| SR7 | pSRC18 | GU433064 | 366 | Heart | 1) Equus caballus gene for beta-myosin heavy chain, partial cds  2) Canis familiaris slow myosin heavy chain beta gene, complete cds | D84227.1  DQ227285.1 | mRNA =1587  mRNA =25235 | --  8 | 639-986  3283-3626 | 251  221 | 94%  93% | 1e-63  2e-54 | 76%  75% |
| SR8 | pSRC19 | GU433065 | 381 | Heart | 1) Homo sapiens 3 BAC RP11-484D18 (Roswell Park Cancer Institute Human BAC Library) complete sequence  2) Pan troglodytes BAC clone CH251-421L18 from chromosome unknown, complete sequence | AC023235.24  AC159111.2 | Gene length =169633  Gene Length =158441 | 3  -- | 163066-162794  2729-2775 | 125  48.2 | 70%  11% | 1e-25  0.032 | 72%  85% |
| SR9 | pSRC20 | GU433066 | 474 | Kidney | 1) Ovis aries clone INRA-164H8, complete sequence  2) Bos taurus clone RP42-394P20, complete sequence  3) Bos taurus Y Chr BAC CH240-477C7 (Children's Hospital Oakland Research Institute Bovine BAC Library (male)) complete sequence | EU185098.1  AC092496.3  AC220322.5 | Gene length =150995  Gene length =216538  Gene length =188436 | --  --  Y | 48856-48734  85404-85305  40056-39945 | 152  148  141 | 26%  21%  23% | 2e-33  2e-32  3e-30 | 95%  100%  92% |
| SR10 | pSRC22 | GU433068 | 493 | Liver | 1) Bos taurus suppressor of Ty 5 homolog (S. cerevisiae), mRNA (cDNA  2) PREDICTED: Equus caballus suppressor of Ty 5 homolog (S. cerevisiae) (SUPT5H), mRNA  3) PREDICTED: Equus caballus suppressor of Ty 5 homolog (S. cerevisiae) (SUPT5H), mRNA | BC134446.1  XM_001915937.1  XM_001088710.1 | mRNA =3590  mRNA =3695  mRNA =3706 | --  10  19 | 2099-2580  2220-2701  2239-2710 | 846  669  662 | 97%  97%  95% | 0.0  0.0  0.0 | 98%  91%  91% |
| SR11 | pSRC23 | GU433069 | 415 | Liver | 1) Human chromosome 14 DNA sequence BAC R-388E23 of library RPCI  2) Anopheles gambiae str. PEST AGAP007820-PA (AgaP_AGAP007820  3) Zebrafish DNA sequence from clone DKEY-184M12 in linkage group | AL160237.4  XM_001688999.1  CR385028.15 | Gene length =206533  mRNA =399  Gene length =185870 | 14  3R  11 | 132269-132022  307-339  127810-127842 | 104  42.8  42.8 | 56%  7%  8% | 5e-19  1.5  1.5 | 70%  87%  88% |
| SR12 | pSRC24  pSRC25 | GU433070  GU433071 | 378  378 | Liver  Spleen | 1) PREDICTED: Bos taurus similar to StAR-related lipid transfer protein 3 (StARD3) (START domain-containing protein 3) (Metastatic lymph node protein 64) (Protein MLN 64) (Protein CAB1), transcript variant 2 (STARD3), mRNA  2) Sus scrofa StAR-related lipid transfer (START) domain containing 3 (STARD3), mRNA  3) PREDICTED: Equus caballus similar to StAR-related lipid transfer protein 3 (StARD3) (START domain-containing protein 3) (Metastatic lymph node protein 64) (Protein MLN 64) (Protein CAB1) (LOC100054870), mRNA | XM_869378.3  NM_001143725.1  XM_001501181.1 | mRNA =1941  mRNA =2092  mRNA =1994 | 19  --  11 | 935-1305  834-1199  745-1110 | 623  503  481 | 97%  96%  96% | 2e-175  3e-139  1e-132 | 97%  91%  90% |
| SR13 | pSRC26 | GU433072 | 306 | Liver | 1) Homo sapiens chromosome 8, clone CTD-2373N4, complete sequence  2) Mus musculus BAC clone RP24-556A11 from chromosome 12, complete sequence | AC102945.2  AC154831.2 | Gene Length =127677  Gene length =199712 | 8  12 | 29105-29032  40421-40383 | 68.0  46.4 | 23%  12% | 3e-08  0.088 | 81%  87% |
| SR14 | pSRC27 | GU433073 | 366 | Liver | 1) Homo sapiens genomic DNA, chromosome 11q, clone:RP11-626H12  2) Equus caballus microsatellite DNA, locus ABGe4441  3) Zebrafish DNA sequence from clone DKEY-246B13, complete sequence | AP003555.2  FN408316.1  CR926461.10 | Gene length =121600  mRNA =282  Gene length =164056 | 11q  8  -- | 48731-48953  109-244  141346-141474 | 116  91.5  86.0 | 57%  36%  34% | 7e-23  3e-15  1e-13 | 70%  75%  75% |
| SR15 | pSRC28  pSRC29 | GU433074  GU433075 | 537  534 | Lung  Spleen | 1) PREDICTED: Canis familiaris similar to G protein-coupled receptor 175 (LOC484621), mRNA  2) Homo sapiens transmembrane protein, adipocyte asscociated 1 (TPRA1  3) PREDICTED: Pan troglodytes G protein-coupled receptor 175, transcript variant 9 (GPR175), mRNA | XM_541735.2  NM_016372.2  XM_001137678.1 | mRNA =1855  mRNA =1873  mRNA =1956 | 20  3q21.2  3 | 266-791  557-1083  643-1169 | 701  636  636 | 97%  98%  98% | 0.0  4e-179  4e-179 | 90%  88%  88% |
| SR16 | pSRC30 | GU433076 | 581 | Lung | 1) Bos taurus complement component 5a receptor 1 (C5AR1), mRNA >gb|AY540054.4| Bos taurus C5a anaphylatoxin receptor mRNA, complete cds  2) Canis lupus familiaris complement component 5a receptor 1 (C5AR1), mRNA >emb|X65860.1| C.familiaris mRNA for complement C5a receptor | NM_001007810.3  NM_001003373.1 | mRNA =1499  mRNA =1993 | 18  1 | 1341-783  1083-747 | 835  268 | 98%  58% | 0.0  2e-68 | 93%  81% |
| SR17 | pSRC31 | GU433077 | 616 | Lung | 1) Ciona intestinalis cDNA, clone:ciad011h06, full insert sequence  2) Entamoeba dispar SAW760 hypothetical protein EDI_049420 mRNA  3) Mus musculus BAC clone RP23-154M23 from chromosome 15, complete | AK173999.1  XM_001736940.1  AC133101.4 | cDNA =3037  cDNA =1251  Gene length =206189 | --  --  15 | 2090-2067  389-431  47522-47556 | 44.6  42.8  42.8 | 3%  7%  5% | 0.66  2.3  2.3 | 100%  82%  88% |
| SR18 | pSRC32  pSRC33 | GU433078  GU433079 | 328  328 | Lung  Ovary | 1) Homo sapiens genomic DNA, chromosome 11q, clone:RP11-626H12  2) Homo sapiens anaplastic lymphoma receptor tyrosine kinase (ALK) on chromosome  3) Myxococcus xanthus DK 1622, complete genome | AP003555.2  NG_009445.1  CP000113.1 | Gene length =121600  Gene length =735793  Gene length =9139763 | 11q  2p23  -- | 48767-48953  28424-28395  6110528-6110587 | 78.8  46.4  46.4 | 53%  9%  17% | 2e-11  0.095  0.095 | 69%  93%  78% |
| SR19 | pSRC34  pSRC34 | GU433080  GU433081 | 326  332 | Testis  Spleen | 1) Bos taurus polymerase (RNA) III (DNA directed) polypeptide C (62kD) (POLR3C), mRNA  2) Sus scrofa mRNA, clone:THY010117G07, expressed in thymus  3) PREDICTED: Equus caballus similar to Polymerase (RNA) III (DNA directed) polypeptide C (62kD) (LOC100065113), mRNA  4) Homo sapiens polymerase (RNA) III (DNA directed) polypeptide C (62kD) (POLR3C), mRNA | NM_001038511.1  AK239631.1  XM_001499277.1  NM_006468.6 | mRNA =2199  mRNA =2213  mRNA =1768    mRNA =1888 | 3  --  5  1q21.1 | 725-1038  714-1024  286-604  370-688 | 564  486  457  418 | 96%  95%  98%  98% | 1e-157  3e-134  2e-125  9e-114 | 99%  94%  92%  90% |
| SR20 | pSRC36 | GU433082 | 450 | Spleen | 1) Bos taurus arrestin, beta 1 (ARRB1), mRNA  2) Sus scrofa mRNA, clone:THY010096B07, expressed in thymus  3) Homo sapiens arrestin, beta 1 (ARRB1), transcript variant 2, mRNA | NM_174243.2  AK239353.1  NM_020251.2 | mRNA =1945  mRNA =1703  mRNA =2180 | 15q25  --  11q13 | 1118-1534  1202-1632  1202-1636 | 737  597  560 | 92%  94%  96% | 0.0  2e-167  2e-156 | 98%  91%  90% |
| SR21 | pSRC37 | GU433083 | 426 | Spleen | 1) Bos taurus zinc finger protein 740 (ZNF740), mRNA  2) PREDICTED: Equus caballus similar to zinc finger protein 740 (LOC100063712), mRNA  3) Homo sapiens zinc finger protein 740 (ZNF740), mRNA | NM_001103252.1  XM_001494843.2  NM_001004304.3 | mRNA =2455  mRNA =1133  mRNA =4256 | 5  6  12q13.13 | 343-754  692-1110  643-1064 | 756  673  641 | 96%  98%  99% | 0.0  0.0  0.0 | 99%  95%  94% |
| SR22 | pSRC38 | GU433084 | 447 | Spleen | 1) Homo sapiens nephronophthisis 4 (NPHP4) on chromosome 1  2) Canis lupus familiaris nephroretinin (NPHP4) gene, NPHP4-e11A allele  3) PREDICTED: Pan troglodytes similar to nephronophthisis 4 (LOC457887 | NG_011724.1  EU707782.1  XR_024323.1 | Gene length =136662  mRNA =562  mRNA =5005 | 1p36.22  5  1 | 69610-69863  236-417  1579-1720 | 194  140  125 | 58%  40%  31% | 4e-46  8e-30  2e-25 | 77%  76%  79% |
| SR23 | pSRC39 | GU433085 | 356 | Spleen | 1) Bos taurus ubiquilin 4 (UBQLN4), mRNA >gb|BC126744.1| Bos taurus ubiquilin 4, mRNA  2) PREDICTED: Pan troglodytes similar to Ubiquilin 4 (LOC736474), mRNA  3) Homo sapiens ubiquilin 4 (UBQLN4), mRNA | NM_001080295.1  XR_020026.1  NM_020131.3 | mRNA =1446  mRNA =3588  mRNA =3572 | 3  3  1q21 | 1-348  68-414  69-415 | 632  536  536 | 97%  97%  97% | 3e-178  3e-149  3e-149 | 99%  94%  94% |
| SR24 | pSRC40 | GU433086 | 247 | Spleen | 1) Mus musculus high mobility group AT-hook 2, mRNA  2) Mus musculus BAC clone RP24-82J4 from chromosome 7, complete sequence  3) Pan troglodytes BAC clone CH251-562F21 from chromosome 17, complete sequence | BC085085.1  AC148972.4  AC183963.2 | mRNA =1848  Gene length =221912  Gene length =213058 | --  7  17 | 1847-1811  17756-17792  203430-203384 | 62.6  59.0  57.2 | 14%  14%  17% | 9e-07  1e-05  4e-05 | 97%  94%  87% |
| SR25 | pSRC41 | GU433087 | 616 | Spleen | 1) Homo sapiens chromosome 9 open reading frame 25, mRNA (cDNA clone MGC:44061 IMAGE:5284905), complete cds  2) MACACA MULATTA BAC clone CH250-425I7 from chromosome 15, complete sequence | BC041009.2  AC201853.3 | mRNA =3567  Gene length =175480 | 9  15 | 2370-3004  112051-111416 | 361  354 | 96%  96% | 2e-96  3e-94 | 72%  72% |
| SR26 | pSRC42 | GU433088 | 565 | Spleen | 1) Bos taurus ATP-binding cassette, sub-family D (ALD), member 1 (ABCD1), mRNA  2) PREDICTED: Equus caballus similar to ATP-binding cassette sub-family D member 1 (Adrenoleukodystrophy protein) (ALDP) (LOC100058868), mRNA  3) Homo sapiens ATP-binding cassette, sub-family D (ALD), member 1 | NM_001046190.1  XM_001491684.1  NM_000033.3 | mRNA =3563  mRNA =2199  mRNA =3697 | X  X  Xq28 | 1872-2417  1470-2016  1860-2406 | 948  756  728 | 96%  96%  96% | 0.0  0.0  0.0 | 97%  91%  90% |
| SR27 | pSRC43 | GU433089 | 486 | Spleen | 1) PREDICTED: Bos taurus similar to interferon regulatory factor 2 binding protein 2 (LOC781665), partial mRNA  2) Human DNA sequence from clone RP4-781K5 on chromosome 1q42.1-43, complete sequence  3) PREDICTED: Equus caballus similar to interferon regulatory factor 2 binding protein 2 (LOC100059147), mRNA | XM_001788085.1  AL160408.24  XM_001916562.1 | mRNA =3983  Gene length =132887  mRNA =4122 | 28  1q42.1-43  1 | 663-815  3136-2901  506-668 | 302  270  257 | 33%  48%  33% | 2e-78  4e-69  3e-65 | 100%  87%  95% |
| SR28 | pSRC44 | GU433090 | 494 | Spleen | 1) Bos taurus Rho-related BTB domain containing 2 (RHOBTB2), mRNA >  2) PREDICTED: Sus scrofa similar to Rho-related BTB domain-containing protein 2 (Deleted in breast cancer 2 gene protein) (p83) (LOC100155280), mRNA  3) PREDICTED: Equus caballus similar to Rho-related BTB domain containing 2 (LOC100057764), mRNA | NM_001103104.1  XM_001929019.1  XM_001490993.2 | mRNA =3136  mRNA =3394  mRNA =2732 | 8  14  2 | 54-488  345-779  347-781 | 780  691  652 | 87%  87%  87% | 0.0  0.0  0.0 | 99%  95%  93% |
| SR29 | pSRC45 | GU433091 | 674 | Testis | 1) PREDICTED: Bos taurus similar to Testis-specific Y-encoded protein 1 (Cancer/testis antigen 78) (CT78) (LOC786811), partial mRNA  2) Bos taurus Y Chr CH240-127C20 (Children's Hospital Oakland Research | XM_001254382.2  AC234853.4 | mRNA =1064  Gene length =130178 | Y  Y | 383-1041  4954-5212 | 1068  396 | 97%  88% | 0.0  1e-106 | 95%  98% |
| SR30 | pSRC46 | GU433092 | 654 | Testis | 1) Homo sapiens UDP-Gal:betaGlcNAc beta 1,4- galactosyltransferase, polypeptide 1 (B4GALT1) on chromosome 9  2) Mouse DNA sequence from clone RP23-135L5 on chromosome 4 Contains the 5' end of the Aptx gene for aprataxin. | NG_008919.1  AL833775.7 | Gene length =63718  Gene length =194640 | 9p13  4 | 34536-34008  138163-138279 | 266  48.2 | 79%  18% | 1e-67  0.058 | 72%  70% |
| SR31 | pSRC47 | GU433093 | 729 | Testis | 1) Pig DNA sequence from clone CH242-243E2 on chromosome X, complete sequence  2) Pan troglodytes chromosome X clone CH251-225C3 map human ortholog p11.4, complete sequence  3) Homo sapiens ornithine carbamoyltransferase (OTC) on chromosome X | CU633374.8  AC197892.2  NG_008471.1 | Gene length =160908  Gene length =164961  Gene length =75968 | X  human ortholog p11.4  Xp21.1 | 45393-44822  107647-107122  3392-2867 | 320  140  136 | 78%  67%  67% | 7e-84  1e-29  2e-28 | 72%  64%  64% |
| SR32 | pSRC48 | GU433094 | 327 | Testis | 1) PREDICTED: Bos taurus similar to polyhomeotic 1-like (PHC1), mRNA  2) PREDICTED: Canis familiaris similar to polyhomeotic 1-like, transcript variant 3 (LOC610167), mRNA  3) PREDICTED: Equus caballus similar to polyhomeotic 1-like, transcript variant 1 (LOC100053465), mRNA  4) Homo sapiens polyhomeotic homolog 1B (Drosophila) (PHC1B) pseudogene on chromosome 12 | XM_590962.4  XM_861897.1  XM_001498983.2  NG_009754.1 | mRNA =3819  mRNA =3294  mRNA =4023  Gene length =5458 | 5  27  6  12q13.2 | 898-1214  1044-1360  936-1252  1202-1518 | 558  448  425  409 | 96%  96%  96%  96% | 5e-156  1e-122  6e-116  6e-111 | 98%  92%  90%  90% |
| SR33 | pSRC49 | GU433095 | 381 | Testis | 1) Homo sapiens 3 BAC RP11-484D18 (Roswell Park Cancer Institute Human BAC Library) complete sequence  2) Pan troglodytes BAC clone CH251-421L18 from chromosome unknown, complete sequence  3) Homo sapiens chromosome 17, clone RP11-1094M14, complete sequence | AC023235.24  AC159111.2  AC015911.8 | Gene length =169633  Gene length =158441  Gene length =181561 | 3  --  17 | 164794-163066  2775-2729  54176-54130 | 125  48.2  48.2 | 70%  11%  11% | 1e-25  0.032  0.032 | 72%  85%  85% |
| SR34 | pSRC50 | GU433096 | 429 | Testis | 1) Lotus japonicus genomic DNA, clone: LjT24A04, TM1735, complete sequence  2) Human DNA sequence from clone RP1-180M12 on chromosome 22 Contains part of the DEPDC5 gene for DEP domain containing 5 and a novel pseudogene similar to e(y)2 protein, complete sequence.  3) M.truncatula DNA sequence from clone MTH2-61P4 on chromosome | AP009699.1  Z82190.1  CT867975.8 | Gene length =65620  Gene length =59941  Gene length =89216 | --  22  3 | 12513-12420  7896-7821  4047-3951 | 93.3  66.2  60.8 | 21%  17%  21% | 1e-15  1e-07  6e-06 | 81%  80%  74% |
| SR35 | pSRC51 | GU433097 | 533 | Testis | 1) Bos taurus BAC CH240-43C16 (Children's Hospital Oakland Research Institute Bovine BAC Library complete sequence  2) Bos taurus SHROOM2 (SHROOM2) gene, partial cds; pseudoautosomal boundary and pseudoautosomal region genomic sequences; and G-protein coupled receptor 143 (GPR143) gene, partial cds, alternatively spliced | AC149676.4  FJ195365.1 | Gene length =168416  Gene length =156628 | --  X | 11715-11541  94619-94775 | 191  185 | 36%  33% | 4e-45  2e-43 | 86%  88% |
| SR36 | pSRC53 | GU433099 | 535 | Testis | 1) Bos taurus clone RP42-194K3, complete sequence  2) Bos taurus clone RP42-341K3, complete sequence | AC089991.2  AC090031.2 | Gene length =159196  Gene length =213841 | --  -- | 33112-32910  176211-176389 | 148  145 | 37%  31% | 2e-32  3e-31 | 80%  97% |
| SR37 | pSRC54 | GU433100 | 342 | Testis | 1) Bos taurus latrophilin-1 type aa mRNA, complete cds  2) PREDICTED: Pan troglodytes similar to KIAA0821 protein (LOC455777), mRNA  3) Homo sapiens latrophilin 1 (LPHN1), transcript variant 1, mRNA | AF111097.1  XR_024518.1  NM_001008701.2 | mRNA =5161  mRNA =8331  mRNA =7874 | --  19  19p13.2 | 3681-3356  4007-3682  3548-3223 | 558  481  475 | 95%  95%  95% | 6e-156  1e-132  6e-131 | 97%  93%  92% |
| SR38 | pSRC55 | GU433101 | 464 | Testis | 1) Homo sapiens SZ-1 mRNA, complete sequence  2) Homo sapiens genomic DNA, chromosome 11q clone:CTD-2537O9, complete sequences | AF525782.1  AP001825.4 | mRNA =10156  Gene length =206624 | 11q14  11q | 4758-4785  189318-189345 | 52.8  52.8 | 6%  6% | 0.002  0.002 | 100%  100% |

1. mRNA transcripts uncovered from spermatozoa.

| Transcript ID | Clone ID | Accession no. | Size(in bp) | Tissue Origin | Details of homology status | Accession no of similar genes | Gene length(bp) | Chromosomal location | Position of (TGG)_5_ tagged transcript | Score | QC% | E-value | Similarity % |
| --- | --- | --- | --- | --- | --- | --- | --- | --- | --- | --- | --- | --- | --- |
| RS1 | pRSC1 | GU391953 | 613 | Semen | 1) Bos taurus BAC CH240-448P19 (Children's Hospital Oakland Research  2) Bos taurus CD4 molecule (CD4), mRNA | AC150635.6  NM_001103225.1 | Gene length =151766  mRNA =2988 | --  5 | 29995-30184  2079-1889 | 189  169 | 31%  30% | 2e-44  2e-39 | 97%  83% |
| RS2 | pRSC2 | GU391954 | 518 | Semen | 1) Bos taurus BAC CH240-105A12 (Children's Hospital Oakland Research Institute Bovine BAC Library (male)) complete sequence  2) Ovis aries clone CH243-50P7, complete sequence  3) Bos taurus chromosome 9 clone BAC RPCI42-522F04, complete sequence | AC150532.4  AC147844.3  DQ405274.1 | Gene length =186851  Gene length =156221  Gene length =202200 | --  --  9 | 7847-7463  7903-8289  35939-36320 | 436  431  425 | 74%  75%  74% | 4e-119  2e-117  9e-116 | 87%  86%  86% |
| RS3 | pRSC3 | GU391955 | 556 | Semen | 1) Bos taurus BTA01 scaffold130001_59457 genomic sequence contig containing highly polymorphic single nucleotide sites  2) Bos taurus BAC CH240-238N22 (Children's Hospital Oakland Research Institute Bovine BAC Library complete sequence  3) Bos taurus phosphatidylinositol transfer protein membrane-associated 2 (PITPNM2) gene, exons 2 through 16, 18 through 23, 25 and partial cds | DQ404150.2  AC150573.6  FJ861208.2 | Gene length =5376  Gene length =163445  Gene length =14989 | 1  --  -- | 3403-3593  65783-65969  945-761 | 213  211  207 | 34%  37%  33% | 8e-52  3e-51  4e-50 | 87%  87%  87% |
| RS4 | pRSC4 | GU391956 | 423 | Semen | 1) Bos taurus clone RP42-351K5, complete sequence  2) Bos taurus BAC CH240-105M18 (Children's Hospital Oakland Research Institute Bovine BAC Library (male)) complete sequence  3) Bos taurus SHROOM2 (SHROOM2) gene, partial cds; pseudoautosomal boundary and pseudoautosomal region genomic sequences; and G-protein coupled receptor 143 (GPR143) gene, partial cds, alternatively spliced | AC092727.2  AC150499.5  FJ195365.1 | Gene length =163604  Gene length =205738  Gene length =156628 | --  Y  X | 147453-147664  100162-100323  27209-27353 | 174  172  163 | 49%  38%  34% | 3e-40  1e-39  6e-37 | 82%  86%  86% |
| RS5 | pRSC5 | GU391957 | 376 | Semen | 1) Bos taurus BAC CH240-493H15 (Children's Hospital Oakland Research Institute Bovine BAC Library (male)) complete sequence  2) Bos taurus BAC CH240-423C21 (Children's Hospital Oakland Research Institute Bovine BAC Library complete sequence  3) Ovis aries clone INRA-164H8, complete sequence | AC149683.2  AC149694.4  EU185098.1 | Gene length =176832  Gene length =186118  Gene length =150995 | --  --  -- | 27089-26888  138108-137909  39929-40120 | 207  195  189 | 54%  54%  55% | 3e-50  2e-46  9e-45 | 95%  87%  92% |
| RS6 | pRSC6 | GU391958 | 403 | Semen | 1) PREDICTED: Bos taurus similar to EH domain-binding protein 1 (EHBP1), partial mRNA  2) Homo sapiens BAC clone RP11-511I11 from 2, complete sequence | XM_612459.3  AC009501.4 | mRNA =2131  Gene length =157173 | 11  2 | 1264-1039  41969-41593 | 398  255 | 56%  90% | 2e-107  1e-64 | 98%  79% |
| RS7 | pRSC7 | GU391959 | 369 | Semen | 1) Human DNA sequence from clone RP11-113P14 on chromosome 13 Contains the 3' end of the DCAMKL1 gene for doublecortin and CaM kinase-like 1 (DCLK KIAA0369), complete sequence  2) Human DNA sequence from clone RP1-89D4 on chromosome 11p12-14.1, complete sequence | AL157760.9  AL136126.34 | Gene length =108649  Gene length =78947 | 13  11p12-14.1 | 107294-107524  37023-37071 | 150  53.6 | 62%  13% | 4e-33  7e-04 | 76%  85% |
| RS8 | pRSC8 | GU391960 | 341 | Semen | 1) Populus trichocarpa predicted protein, mRNA  2) Agkistrodon contortrix clone E33DIFJ02I9LCK microsatellite sequence | XM_002313503.1  GQ190354.1 | mRNA =1060  Gene length =247 | --  -- | 677-650  65-88 | 48.8  46.8 | 8%  7% | 0.020  0.075 | 96%  100% |
| RS9 | pRSC9 | GU391961 | 305 | Semen | 1) Conexibacter woesei DSM 14684, complete genome  2) Canis lupus familiaris clone RP81-117B1, complete sequence | CP001854.1  AC091624.3 | Gene length =6359369  Gene length =174712 | --  -- | 28500-28479  172269-172296 | 43.0  43.0 | 7%  9% | 0.95  0.95 | 100%  92% |
| RS10 | pRSC10 | GU391962 | 275 | Semen | 1) Pig DNA sequence from clone CH242-227A13 on chromosome 14, complete sequence  2) Homo sapiens PAC clone RP1-130H16 from 22q12.1-qter, complete sequence | CT827833.7  AC004997.2 | Gene length =165239  Gene length =109238 | 14  22q12.1-qter | 145436-145225  21790-21663 | 166  110 | 78%  46% | 8e-38  5e-21 | 81%  82% |
| RS11 | pRSC11 | GU391963 | 295 | Semen | 1) Cryptococcus neoformans var. neoformans JEC21 chromosome 7, complete sequence  2) Mus musculus clone 11AS1-1 LINE L1 ASL1/Dzip3 fusion mRNA sequence | AE017347.1  EU233997.1 | Gene length =1347793  mRNA =3343 | 7  -- | 994015-993987  2441-2468 | 44.9  43.0 | 9%  9% | 0.25  0.93 | 93%  92% |
| RS12 | pRSC12 | GU391964 | 261 | Semen | 1) Bos taurus clone RP42-139G14, complete sequence  2) Bos taurus glutamate-cysteine ligase catalytic subunit (GCLC) | AC146966.3  AY957499.1 | Gene length =179474  mRNA =447010 | --  -- | 115526-115640  123343-123230 | 196  195 | 44%  45% | 4e-47  4e-47 | 97%  97% |
| RS13 | pRSC13 | GU391965 | 236 | Semen | 1) Pan troglodytes BAC clone RP43-10J15 from chromosome 7, complete sequence  2) Homo sapiens chromosome 7 clone RP11-377H23, complete sequence | AC145921.3    AC078841.4 | Gene length =201403  Gene length =187497 | 7  7 | 98916-98853  88385-88446 | 62.6  62.6 | 27%  26% | 9e-07  9e-07 | 81%  82% |
| RS14 | pRSC14 | GU391966 | 216 | Semen | 1) Homo sapiens chromosome 4 clone C0548P18 map 4p16, complete sequence  2) Mus musculus BAC clone RP24-341K21 from chromosome 10, complete sequence | AC006568.7  AC144852.3 | Gene length =188705  Gene length =169565 | 4p16  10 | 28349-28517  127057-127084 | 127  42.8 | 77%  12% | 2e-26  0.73 | 77%  92% |
| RS15 | pRSC15 | GU391967 |  | Semen | 1) Pongo abelii BAC clone CH276-13H12 from chromosome unknown, complete sequence  2) Pan troglodytes BAC clone CH251-534A21 from chromosome x, complete sequence | AC190402.1  AC185343.2 | Gene length =220378  Gene length =183024 | --  X | 151482-151524  8636-8685 | 57.2  51.8 | 16%  19% | 4e-05  0.002 | 90%  84% |
