# Supplementary material for: Molecular Mining of Alleles in Water Buffalo Bubalus bubalis and Characterization of the TSPY1 and COL6A1 Genes
Source: PLoS One. 2011 Sep 15;6(9):e24958. doi: 10.1371/journal.pone.0024958 (PMC3174239; doi:10.1371/journal.pone.0024958)
Supplement: Table S3 — List of primers used for RT-PCR on cDNA from different tissues (i) and semen (ii). The primer IDs and corresponding gene accession number of the amplified transcripts are given in the table. (DOC) [file pone.0024958.s005.doc]

**Table S3:**

**(i) Details of the primers used for RT-PCR with cDNA from different tissues**

| **S.No.** | **Oligo ID** | **Accession no.** | **Sequence (5’-3’)** | **Annealing Temp.**  **(°C)** |
| --- | --- | --- | --- | --- |
| 1. | SA1668 | GU433047 | F CAAGGCGGCTGAGTATGAC | 60 |
|  | SA1669 |  | R TGACCCCAGAAAGGACAGAG |  |
| 2. | SA1670 | GU433054 | F ACTACGACTTGCGCCACACT | 60 |
|  | SA1671 |  | R CCATGACAATCAAGCTGCTG |  |
| 3. | SA1674 | GU433059 | F AGCAGCAGGGACAGACAGAT | 60 |
|  | SA1675 |  | R GGGGACACACGAGTTCAGTT |  |
| 4. | SA1676 | GU433061 | F GAGAAGAGGAAGCACGCAGT | 60 |
|  | SA1677 |  | R CTTCTGACCCCAGAACCTTG |  |
| 5. | SA1678 | GU433062 | F TGTAACTTGCCCCCTTTGAC | 60 |
|  | SA1679 |  | R GGAGAACGGAAAAGAGCAGA |  |
| 6. | SA1680 | GU433063 | F AGGAGCTGGAAAGGGAAGAG | 60 |
|  | SA1681 |  | R TGCAGATCTGAGTTCGCTGA |  |
| 7. | SA1682 | GU433064 | F GCCAGAGCAAGCTGTTAGGT | 60 |
|  | SA1683 |  | R GGACCCCAGAAAAGGGAATA |  |
| 8. | SA1684 | GU433065 | F GGAGGAAGTGCAAAGACAGC | 60 |
|  | SA1685 |  | R GTGGCAAGATTTGTGGAGGT |  |
| 9. | SA1686 | GU433066 | F GTGGGACAGAGGCTCAAGAA | 60 |
|  | SA1687 |  | R CTGCCAATGCAGGTTAGACA |  |
| 10. | SA1688 | GU433068 | F ACCTGCCAGACCATCTCAGT | 60 |
|  | SA1689 |  | R GGGGTAGGCTCATCATCAAA |  |
| 11. | SA1690 | GU433069 | F GGGCTCTGCTCTGAGTTGAA | 60 |
|  | SA1691 |  | R CGTCCTTTGGACAATCTTGG |  |
| 12. | SA1692 | GU433070 | F GCCCAAGAAGAGAACTGGAA | 60 |
|  | SA1693 |  | R ATGCCCGAGGACAGGTATC |  |
| 13. | SA1694 | GU433072 | F GCGTCTGCTGGTTCTCTTTC | 60 |
|  | SA1695 |  | R AGCACTTGGCACAGAAAACA |  |
| 14. | SA1696 | GU433073 | F GGATGAACAGAGGATGGAGTG | 60 |
|  | SA1697 |  | R GATGGGGAAGGTGGTAGAGG |  |
| 15. | SA1698 | GU433074 | F AGATCCTGTGGGAGATCACG | 60 |
|  | SA1699 |  | R CAGAGCCCCTCAATGATGTC |  |
| 16. | SA1700 | GU433076 | F GGATGATGCTGGCCTTTTT | 60 |
|  | SA1701 |  | R GGGGGCAGTCCTAGAAGAAC |  |
| 17. | SA1702 | GU433077 | F CTGGAATTTGAGAGGCCTGA | 60 |
|  | SA1703 |  | R CCAGGGATGACCTTACCACA |  |
| 18. | SA1704 | GU433078 | F GTGGATGAATGGAGCATGG | 60 |
|  | SA1705 |  | R ATGGGCAGAACCCCTGAT |  |
| 19. | SA1706 | GU433080 | F GTATGAAGCCCAGTGCAACC | 60 |
|  | SA1707 |  | R GCTGCACAAAGTGTGTGTCC |  |
| 20. | SA1708 | GU433082 | F TGGAACTGCCTTTCACCCTA | 60 |
|  | SA1709 |  | R CACCGTGACCTGGAAGACC |  |
| 21. | SA1710 | GU433083 | F GAACACTGCTTTGGAGCCTTT | 60 |
|  | SA1711 |  | R GTGGGACCATCAGACCAGAG |  |
| 22. | SA1712 | GU433084 | F ACGGGAGCAATGTTGTTGAT | 60 |
|  | SA1713 |  | R TGCATTCTTTGTGGGTGTGT |  |
| 23. | SA1714 | GU433085 | F ATTCGGGTCACCGTCAAGA | 60 |
|  | SA1715 |  | R CTGGGTCTGGGGTGGAAG |  |
| 24. | SA1716 | GU433086 | F CCCATTGCCAAGAATGACTT | 60 |
|  | SA1717 |  | R GAAGGTGTAGGCAGGTGAGG |  |
| 25. | SA1718 | GU433087 | F GCCAGAAAAGCTGAGGATGA | 60 |
|  | SA1719 |  | R AGTAGGAAGCATGGCTCTGG |  |
| 26. | SA1720 | GU433088 | F GTGGCCAGTCTCAACATCAG | 60 |
|  | SA1721 |  | R CTTCCACGTCGATGCTCAC |  |
| 27. | SA1722 | GU433089 | F AGGGCAGCCTGGGTACTT | 60 |
|  | SA1723 |  | R GGCGACACAAAAGAGGATGT |  |
| 28. | SA1860 | GU433090 | F GGCAAGACCAGGCTTATCTG | 60 |
|  | SA1861 |  | R GTCTCCTGGCCCTATTGACA |  |
| 29. | SA1660 | GU433091 | F CATACGAGGAGCAGGAGCAG | 60 |
|  | SA1661 |  | R GCCACAGGTCCTTTATGATGA |  |
| 30. | SA1726 | GU433092 | F CCATAGGCCTGGATTACTCG | 60 |
|  | SA1727 |  | R AGTTCCAACCCCTGGAATTT |  |
| 31. | SA1862 | GU433093 | F ACGAGATGGCAGCTCTGTTT | 60 |
|  | SA1863 |  | R GATGGCAAACCCTGTATGCT |  |
| 32. | SA1730 | GU433094 | F GGCAGTGACTGTGAGTCAGG | 60 |
|  | SA1731 |  | R CTGGATCACCACCTGCTTCT |  |
| 33. | SA1732 | GU433095 | F GTGGCAAGATTTGTGGAGGT | 60 |
|  | SA1733 |  | R CGTTCTGCTGACACATCTGG |  |
| 34. | SA1734 | GU433096 | F CACAGACCTGTGTGCAGTCAC | 60 |
|  | SA1735 |  | R GAAGACAAAGCCCTCCATGA |  |
| 35. | SA1736 | GU433097 | F CGACGTGCCAATGTAAGAGA | 60 |
|  | SA1737 |  | R TTACAAAGGTGCAGGCAGAA |  |
| 36. | SA1716 | GU433098 | F CCCATTGCCAAGAATGACTT | 60 |
|  | SA1717 |  | R GAAGGTGTAGGCAGGTGAGG |  |
| 37. | SA1738 | GU433099 | F GGTGGGGAGAGGTTAAGGAA | 60 |
|  | SA1739 |  | R ATCCCCTAGAGGAGGGCATA |  |
| 38. | SA1740 | GU433100 | F CCATCGACTACCGCAGCTA | 60 |
|  | SA1741 |  | R CACGGACTCCTTGTTGATGA |  |
| 39. | SA1864 | GU433101 | F CTTGGCTGGGCTAAGAGATG | 60 |
|  | SA1865 |  | R GTCCAGAGGCCTGAGAACTG |  |
| 40. | BBACTF | BETA ACTIN | F CAGATCATGTTCGAGACCTTCAA | 60 |
|  | BBACTR |  | R GATGATCTTGATCTTCATTGTGCTG |  |

**(ii)** Details of the primers used for RT-PCR with cDNA from the semen samples

| **S.No.** | **Oligo ID** | **Accession no.** | **Sequence (5’-3’)** | **Annealing Temp.**  **(°C)** |
| --- | --- | --- | --- | --- |
| 1. | SA1957 | GU391953 | F TGAAGCCCCATGGACTGTAG | 60 |
|  | SA1958 |  | R GTGGGGTCATCTAGGAGTCG |  |
| 2. | SA1959 | GU391954 | F CTTGTTCAGGCATGATGTGG | 60 |
|  | SA1960 |  | R GGCAGTCCTAAGATGGTGGA |  |
| 3. | SA1961 | GU391955 | F GCTCCTGCCCTGATATGAAA | 60 |
|  | SA1962 |  | R CCAGGCTCCTCTGTCCATAA |  |
| 4. | SA1963 | GU391956 | F CCTATCAATGCTGGGGTCCT | 60 |
|  | SA1964 |  | R GTGTCTGCTGGTCACTGAGG |  |
| 5. | SA1965 | GU391957 | F CGGGAGACATTTGACAGTGTT | 60 |
|  | SA1966 |  | R CTGCCAATGCAGGAGATGTA |  |
| 6. | SA1967 | GU391958 | F CAATCCAGTCAAACAAAATGTTAAA | 60 |
|  | SA1968 |  | R GGTCGGGGATTTCTATCCAG |  |
| 7. | SA1969 | GU391959 | F TGAAGGCAAGCAACTCATTG | 60 |
|  | SA1970 |  | R GGGGGTGCAGTTATCATTGT |  |
| 8. | SA1971 | GU391960 | F GGTCTTGGAGAAGCGAATCA | 60 |
|  | SA1972 |  | R CTGCTGAGGTAGCCCATAGC |  |
| 9. | SA1973 | GU391961 | F CTTCCCAATGTCCTCCTCCT | 60 |
|  | SA1974 |  | R GCACACTGGTGTGTCCAAAT |  |
| 10. | SA1975 | GU391962 | F TCCCCACTTATGACTGACAGC | 60 |
|  | SA1976 |  | R AAAGAACCACTGGCATTTTCA |  |
| 11. | SA1977 | GU391963 | F GGCTTGCACCATTAATAAAAGC | 60 |
|  | SA1978 |  | R GGACTGGAAAGAGGAAGATGG |  |
| 12. | SA1979 | GU391964 | F GAACACTCTTGAAAGTCCCTTGA | 60 |
|  | SA1980 |  | R TGTTCTTGCCTGGAGAATCC |  |
| 13. | SA1981 | GU391965 | F GATAGGGGGAGGGGGAGT | 60 |
|  | SA1982 |  | R CAGGAGGCTCCATTACTGCT |  |
| 14. | SA1983 | GU391966 | F GGCAATGCAGAGGCTACAA | 60 |
|  | SA1984 |  | R CAGCAGTGTCACCTATGAGCA |  |
| 15. | SA1716 | GU391967 | F CCCATTGCCAAGAATGACTT | 60 |
|  | SA1717 |  | R GAAGGTGTAGGCAGGTGAGG |  |
