# Supplementary material for: Molecular Mining of Alleles in Water Buffalo Bubalus bubalis and Characterization of the TSPY1 and COL6A1 Genes
Source: PLoS One. 2011 Sep 15;6(9):e24958. doi: 10.1371/journal.pone.0024958 (PMC3174239; doi:10.1371/journal.pone.0024958)
Supplement: Table S4 — List of primers used for q-PCR on cDNA from different tissues (i) and semen samples (ii). (DOC) [file pone.0024958.s006.doc]

**Table S4:**

**(i)** Details of the primers used for relative expressional studies in different tissues

| **S.No.** | **Oligo ID** | **Accession no.** | **Sequence (5’-3’)** | **Annealing Temp.**  **(°C)** |
| --- | --- | --- | --- | --- |
| 1. | SA1782 | GU433047 | F GCCACCGAGACAGATGCAA | 60 |
|  | SA1783 |  | R TCAAGCTTTCCCTTTAAAAAATCC |  |
| 2. | SA1784 | GU433054 | F AGATGAGATGGTTTGGCACAGA | 60 |
|  | SA1785 |  | R CCCCTACCCATACCCATGACT |  |
| 3. | SA1788 | GU433059 | F GGTCACACAAAGAATGGAAAAGC | 60 |
|  | SA1789 |  | R CCTCTGACCCCGTATCTCCTAGT |  |
| 4. | SA1790 | GU433061 | F TCGCATTCTGTCTGAACACTCA | 60 |
|  | SA1791 |  | R GGCCCCCACCCCTGTAT |  |
| 5. | SA1792 | GU433062 | F AGACATGCCCAGAGCACCAT | 60 |
|  | SA1793 |  | R GCAAGGAAATGAGAGGAAAGGA |  |
| 6. | SA1794 | GU433063 | F CGGCGCACAGTCAAGATG | 60 |
|  | SA1795 |  | R TGCCTTCTGCAGGAATTGCT |  |
| 7. | SA1796 | GU433064 | F CATATGAGAGATCCAGGCATGGA | 60 |
|  | SA1797 |  | R CCACTCACGTTTCCTACCACTGT |  |
| 8. | SA1798 | GU433065 | F GGTGGTTAGGAGGAAGTGCAAA | 60 |
|  | SA1799 |  | R AGGCCAGATGTGTCAGCAGAA |  |
| 9. | SA1800 | GU433066 | F TTTTGGCTGATTCGCATTGTT | 60 |
|  | SA1801 |  | R GAGGATAACTGCTTTGCGATGTT |  |
| 10. | SA1802 | GU433068 | F GACCCAAACAACCCCAATACA | 60 |
|  | SA1803 |  | R GCTCATCATCAAAGGCATACTCAT |  |
| 11. | SA1804 | GU433069 | F ATTTGCTGGGCACATTGGTT | 60 |
|  | SA1805 |  | R CCTTTCCACTACCTCCCCTCTAA |  |
| 12. | SA1806 | GU433070 | F CTGCCAGATCCTACAACGAGTAGAG | 60 |
|  | SA1807 |  | R GGCCCCTGAAGACACATCA |  |
| 13. | SA1808 | GU433072 | F GGCTGCCTGCCATTGC | 60 |
|  | SA1809 |  | R CTGCCCTTTCCACCTTTGTC |  |
| 14. | SA1810 | GU433073 | F TTCCTCCAGAACAGTGACACATG | 60 |
|  | SA1811 |  | R CCTCGCCCCTCACTGATG |  |
| 15. | SA1812 | GU433074 | F CGACGGTGGCTGACAAGAT | 60 |
|  | SA1813 |  | R TGACCACGCTCAGCTCGAT |  |
| 16. | SA1814 | GU433076 | F TGCTGCATTAACCCCATCATT | 60 |
|  | SA1815 |  | R GAACCGGGCGTGAAAGC |  |
| 17. | SA1816 | GU433077 | F AACCTTTGCTTCCAATCTTGTCTAG | 60 |
|  | SA1817 |  | R GCTGGCTTGGCAAAAGTAACC |  |
| 18. | SA1818 | GU433078 | F AGGCGCTGGGCAATGA | 60 |
|  | SA1819 |  | R CACTTAGGCCGTTTCCAAACA |  |
| 19. | SA1820 | GU433080 | F GGACTATGCTGAGGTATCAAACACA | 60 |
|  | SA1821 |  | R GGGCAACGCTGCACAAAG |  |
| 20. | SA1822 | GU433082 | F ATGACGACATTGTGTTTGAGGACTT | 60 |
|  | SA1823 |  | R CCTTGTCATCCTTCATGCCTTT |  |
| 21. | SA1824 | GU433083 | F GGAGCCTTTAGGAGCAGTTATCAC | 60 |
|  | SA1825 |  | R AATGGCTTCTCACCGGTATGA |  |
| 22. | SA1826 | GU433084 | F ACCCGCGTCCCCTTCA | 60 |
|  | SA1827 |  | R GCTGTGCCACGAGCTTGA |  |
| 23. | SA1828 | GU433085 | F CATTCGGGTCACCGTCAAG | 60 |
|  | SA1829 |  | R AGGCTCGGTCGCAGATCAC |  |
| 24. | SA1830 | GU433086 | F GGTGGTGGCAGTGGAAGCTA | 60 |
|  | SA1831 |  | R CCTGCGGGTGGAACTGAA |  |
| 25. | SA1832 | GU433087 | F CCAACCCCACCTCCTCTTAGT | 60 |
|  | SA1833 |  | R TCCCTTTGGCATTTCCTCAT |  |
| 26. | SA1834 | GU433088 | F TCGACGTGGAAGGGAAGATC | 60 |
|  | SA1835 |  | R GGTCGGTGGGTGATGGAA |  |
| 27. | SA1836 | GU433089 | F AGGGCAGCCTGGGTACTTG | 60 |
|  | SA1837 |  | R GGAAAATGGCACGGATAGCA |  |
| 28 | SA1838 | GU433090 | F ACAACGCTGTGGGCAAGAC | 60 |
|  | SA1839 |  | R ACTGGGTGAGGGTGGCATT |  |
| 29. | SA1840 | GU433091 | F CCGTGAATAAGGAAGCCCAAA | 60 |
|  | SA1841 |  | R GCCGCTGACAGGTCTTACG |  |
| 30. | SA1842 | GU433092 | F AGGGCATCTGGATTGTCACTCT | 60 |
|  | SA1843 |  | R CCTCTTTCCTGGGTTGGTTTC |  |
| 31. | SA1844 | GU433093 | F GTGGGCAAACTGCACTCAACT | 60 |
|  | SA1845 |  | R GGGTAGCATCCTTGACTGAAGAG |  |
| 32. | SA1846 | GU433094 | F CAACAGAACGTGGGCATGAA | 60 |
|  | SA1847 |  | R GCGTGTAGGTGGCTGAGCTAA |  |
| 33. | SA1848 | GU433095 | F CTCAAAGTCTACAGGACCGGAAA | 60 |
|  | SA1849 |  | R TGCACGTTCTGCTGACACATC |  |
| 34. | SA1850 | GU433096 | F CTGTTAGCTGGGTGTCCTGAGA | 60 |
|  | SA1851 |  | R AGTGAGGGTGCTGAGGCTTGT |  |
| 35. | SA1852 | GU433097 | F AGGAATCGACGTGCCAATG | 60 |
|  | SA1853 |  | R CCGAGCCAGGGATCAAATC |  |
| 36. | SA1830 | GU433098 | F GGTGGTGGCAGTGGAAGCTA | 60 |
|  | SA1831 |  | R CCTGCGGGTGGAACTGAA |  |
| 37. | SA1854 | GU433099 | F TGCGTGCATGAGTGCTAAGTC | 60 |
|  | SA1855 |  | R GGCAGGCTACATTCCATAGCAT |  |
| 38. | SA1856 | GU433100 | F GCCCGTCTCCTTTGTTATTGTG | 60 |
|  | SA1857 |  | R CGGACCACCTTGTGCAGAGT |  |
| 39. | SA1858 | GU433101 | F TGGGCGGGCACTGACT | 60 |
|  | SA1859 |  | R GTCTACGCTTCCCCCATCTTT |  |
| 40. | SAR1651 | GAPDH | F GCAAGTTCCACGGCACAGT | 60 |
|  | SAR1652 |  | R GATGGTGATGGCCTTTCCAT |  |

**(ii)** Details of the primers used for relative expressional studies in the Semen samples

| **S.No.** | **Oligo ID** | **Accession no.** | **Sequence (5’-3’)** | **Annealing Temp.**  **(°C)** |
| --- | --- | --- | --- | --- |
| 1. | SA1883 | GU391953 | F AGTGGGTTGCCATTTCCTTCT | 60 |
|  | SA1884 |  | R CAATGCAGGAGATGTGGGTTT |  |
| 2. | SA1885 | GU391954 | F GGGAGGGAAGAACACTGCAA | 60 |
|  | SA1886 |  | R CTCCCAGCAGCCCAGATG |  |
| 3. | SA1887 | GU391955 | F TCAACCCACCCACATTCATG | 60 |
|  | SA1888 |  | R AGAGGAGCTGGCAAAAAAAGG |  |
| 4. | SA1889 | GU391956 | F AAATGGCAACCCACTCCAGTA | 60 |
|  | SA1890 |  | R AGTTTGCCAGGCTTCTCTGTTTA |  |
| 5. | SA1891 | GU391957 | F CAGTAAGTCGCTTCAGTCGTGTCT | 60 |
|  | SA1892 |  | R CCTGGCGGGTTTACAGTCA |  |
| 6. | SA1893 | GU391958 | F CGGTTTCTACTGAAGGACACATTTC | 60 |
|  | SA1894 |  | R GCGAACTCTGGAACAAAACAAA |  |
| 7. | SA1895 | GU391959 | F ATGCCACTTGCCTTCAGGAT | 60 |
|  | SA1896 |  | R GCCTTTCAGGATGATGCTAATCTC |  |
| 8. | SA1897 | GU391960 | F TGTCAGGTCAATGGCACACA | 60 |
|  | SA1898 |  | R TGAGAAGAGCAGGCAATAGTGTTT |  |
| 9. | SA1899 | GU391961 | F TGCATAGTCCCTACAGCAAACCT | 60 |
|  | SA1900 |  | R TTTCCTTGTGTGCCATGACAGT |  |
| 10. | SA1901 | GU391962 | F CCCACTTATGACTGACAGCAGAGT | 60 |
|  | SA1902 |  | R CGAAGCGTGCAGGCACTAA |  |
| 11. | SA1903 | GU391963 | F TGCCAACAGCGTCTCTCGTA | 60 |
|  | SA1904 |  | R GAAGGCAATAGCGGTGTGTCTA |  |
| 12. | SA1905 | GU391964 | F TTCACCAGAAGAATTGAAGCTGAA | 60 |
|  | SA1906 |  | R CGGACACGACTGAAGCAACTTA |  |
| 13. | SA1907 | GU391965 | F GATTCATGCCATCAGCCAATT | 60 |
|  | SA1908 |  | R TCCCCCCAGCAAGAAAAGA |  |
| 14. | SA1909 | GU391966 | F ACACAGGCAACTTTGTTTCCAA | 60 |
|  | SA1910 |  | R ACCACCTCCAGGAGACACAGAT |  |
| 15. | SA1830 | GU391967 | F GGTGGTGGCAGTGGAAGCTA | 60 |
|  | SA1831 |  | R CCTGCGGGTGGAACTGAA |  |
| 16. | SA1986 | GAPDH | F TGACCCCTTCATTGACCTTC | 60 |
|  | SA1987 |  | R GTCTTCTGGGTGGCAGTGAT |  |
