# Supplementary material for: Molecular Mining of Alleles in Water Buffalo Bubalus bubalis and Characterization of the TSPY1 and COL6A1 Genes
Source: PLoS One. 2011 Sep 15;6(9):e24958. doi: 10.1371/journal.pone.0024958 (PMC3174239; doi:10.1371/journal.pone.0024958)
Supplement: Table S5 — List of primers used for obtaining full length CDS of the COL6A1 gene (i) and TSPY1 -like gene (ii). (DOC) [file pone.0024958.s007.doc]

**Table S5:**

**(i): List of the primers used for obtaining full length CDS of *COL6A1*** gene

| **S. No.** | **Oligo ID** | **Primer sequence 5’-3’** | **Product Size(in bp)**  **cDNA** | **Annealing Temp.**  **(°C)** |
| --- | --- | --- | --- | --- |
| 1. | SA1941  SA1942 | F ATTGCTCCATGGTGACACG  R CCAGGTCTTCCAGGGTCTC | 900 | 60 |
| 2. | SA1943  SA1944 | F GGGTACGAGGGAGAAAGAGG  R GCCTTCAGGACCTCGGTAG | 939 | 57 |
| 3. | SA1945  SA1946 | F CCCGGAGAGGACAATAATGA  R AAGCGTTTGGTGATGTCAAA | 887 | 60 |
| 4. | SA1947  SA1948 | F ACATCACCATCCTCCTGGAC  R TCCCCTCTTGCATCTGTCTC | 641 | 60 |

**(ii): Primer used for retrieving 5’ end of *TSPY1-***like gene

| **S. No.** | **Oligo ID** | **Primer sequence 5’-3’** | **Product Size(in bp)**  **cDNA** | **Annealing Temp.**  **(°C)** |
| --- | --- | --- | --- | --- |
| 1. | SA1666 | TCCAGCTTGTTTGCAGTTCC | - | 60 |
