# Supplementary material for: Molecular Mining of Alleles in Water Buffalo Bubalus bubalis and Characterization of the TSPY1 and COL6A1 Genes
Source: PLoS One. 2011 Sep 15;6(9):e24958. doi: 10.1371/journal.pone.0024958 (PMC3174239; doi:10.1371/journal.pone.0024958)
Supplement: Table S6 — Details of the clones corresponding to different mRNA transcripts uncovered by MASA showing significant homologies with the cDNA sequences in the Database. This table shows details of only 14 mRNA transcripts of the 38 identified from different somatic tissues and gonads. Significantly, all the query sequences, irrespective of their size, showed >95% identity with the sequences in the GenBank. (DOC) [file pone.0024958.s008.doc]

**Table S6: Details of the clones corresponding to different mRNA transcripts uncovered by MASA showing significant homologies with the cDNA sequences in the Database**

| Transcript ID | Clone ID | Accession no. | Size(in bp) | Tissue Origin | Details of homology status | Accession no of similar genes | Gene length(bp) | Chromosomal location | Position of (TGG)_5_ tagged transcript | Score | QC% | E-value | Similarity % |
| --- | --- | --- | --- | --- | --- | --- | --- | --- | --- | --- | --- | --- | --- |
| SR 1 | pSRC1  pSRC2  pSRC3  pSRC4  pSRC5  pSRC6  pSRC7 | GU433047  GU433048  GU433049  GU433050  GU433051  GU433052  GU433053 | 507  507  507  510  507  522  510 | Heart  Kidney  Liver  Lung  Spleen  Testis  Ovary | 1) Bos taurus collagen, type VI, alpha 1 (COL6A1), mRNA  2) PREDICTED: Canis familiaris collagen VI-alpha1 protein (COL6A1), | NM_001143865.1  XM_548729.2 | mRNA =4069  mRNA =621 | 1q12-q14  31 | 3129-3626  445-621 | 824  272 | 97%  34% | 0.0  1e-69 | 96%  94% |
| SR2 | pSRC8  pSRC9  pSRC10  pSRC11  pSRC12 | GU433054  GU433055  GU433056  GU433057  GU433058 | 465  465  465  465  465 | Heart  Liver  Lung  Testis  Ovary | 1) Bos taurus alpha-2-HS-glycoprotein (AHSG), mRNA >emb|X16577.1| B.taurus mRNA for fetuin  2) Ovis aries fetuin (LOC443392), mRNA >emb|X16578.1| Ovis aries mRNA for fetuin | NM_173984.2  NM_001009802.1 | mRNA =1459  mRNA =1518 | 1  - | 882-1337  941-1389 | 813  691 | 97%  97% | 0.0  0.0 | 98%  94% |
| SR6 | pSRC17 | GU433063 | 394 | Heart | 1) Bos taurus family with sequence similarity 50, member A (FAM50A), mRNA  2) PREDICTED: Equus caballus misc_RNA (LOC100058349), miscRNA  3) Homo sapiens family with sequence similarity 50, member A (FAM50A), mRNA | NM_001034496.2  XR_044493.1  NM_004699.2 | mRNA =1517  mRNA =6857  mRNA =1357 | X  X  X | 482-861  430-808  509-884 | 675  534  507 | 96%  96%  95% | 0.0  1e-148  2e-140 | 98%  92%  91% |
| SR10 | pSRC22 | GU433068 | 493 | Liver | 1) Bos taurus suppressor of Ty 5 homolog (S. cerevisiae), mRNA (cDNA  2) PREDICTED: Equus caballus suppressor of Ty 5 homolog (S. cerevisiae) (SUPT5H), mRNA  3) PREDICTED: Equus caballus suppressor of Ty 5 homolog (S. cerevisiae) (SUPT5H), mRNA | BC134446.1  XM_001915937.1  XM_001088710.1 | mRNA =3590  mRNA =3695  mRNA =3706 | --  10  19 | 2099-2580  2220-2701  2239-2710 | 846  669  662 | 97%  97%  95% | 0.0  0.0  0.0 | 98%  91%  91% |
| SR12 | pSRC24  pSRC25 | GU433070  GU433071 | 378  378 | Liver  Spleen | 1) PREDICTED: Bos taurus similar to StAR-related lipid transfer protein 3 (StARD3) (START domain-containing protein 3) (Metastatic lymph node protein 64) (Protein MLN 64) (Protein CAB1), transcript variant 2 (STARD3), mRNA  2) Sus scrofa StAR-related lipid transfer (START) domain containing 3 (STARD3), mRNA | XM_869378.3  NM_001143725.1 | mRNA =1941  mRNA =2092 | 19  -- | 935-1305  834-1199 | 623  503 | 97%  96% | 2e-175  3e-139 | 97%  91% |
| SR15 | pSRC28  pSRC29 | GU433074  GU433075 | 537  534 | Lung  Spleen | 1) PREDICTED: Canis familiaris similar to G protein-coupled receptor 175 (LOC484621), mRNA  2) Homo sapiens transmembrane protein, adipocyte asscociated 1 (TPRA1  3) PREDICTED: Pan troglodytes G protein-coupled receptor 175, transcript variant 9 (GPR175), mRNA | XM_541735.2  NM_016372.2  XM_001137678.1 | mRNA =1855  mRNA =1873  mRNA =1956 | 20  3q21.2  3 | 266-791  557-1083  643-1169 | 701  636  636 | 97%  98%  98% | 0.0  4e-179  4e-179 | 90%  88%  88% |
| SR19 | pSRC34  pSRC34 | GU433080  GU433081 | 326  332 | Testis  Spleen | 1) Bos taurus polymerase (RNA) III (DNA directed) polypeptide C (62kD) (POLR3C), mRNA  2) Sus scrofa mRNA, clone:THY010117G07, expressed in thymus  3) PREDICTED: Equus caballus similar to Polymerase (RNA) III (DNA directed) polypeptide C (62kD) (LOC100065113), mRNA  4) Homo sapiens polymerase (RNA) III (DNA directed) polypeptide C (62kD) (POLR3C), mRNA | NM_001038511.1  AK239631.1  XM_001499277.1  NM_006468.6 | mRNA =2199  mRNA =2213  mRNA =1768    mRNA =1888 | 3  --  5  1q21.1 | 725-1038  714-1024  286-604  370-688 | 564  486  457  418 | 96%  95%  98%  98% | 1e-157  3e-134  2e-125  9e-114 | 99%  94%  92%  90% |
| SR20 | pSRC36 | GU433082 | 450 | Spleen | 1) Bos taurus arrestin, beta 1 (ARRB1), mRNA  2) Sus scrofa mRNA, clone:THY010096B07, expressed in thymus  3) Homo sapiens arrestin, beta 1 (ARRB1), transcript variant 2, mRNA | NM_174243.2  AK239353.1  NM_020251.2 | mRNA =1945  mRNA =1703  mRNA =2180 | 15q25  --  11q13 | 1118-1534  1202-1632  1202-1636 | 737  597  560 | 92%  94%  96% | 0.0  2e-167  2e-156 | 98%  91%  90% |
| SR21 | pSRC37 | GU433083 | 426 | Spleen | 1) Bos taurus zinc finger protein 740 (ZNF740), mRNA  2) PREDICTED: Equus caballus similar to zinc finger protein 740 (LOC100063712), mRNA  3) Homo sapiens zinc finger protein 740 (ZNF740), mRNA | NM_001103252.1  XM_001494843.2  NM_001004304.3 | mRNA =2455  mRNA =1133  mRNA =4256 | 5  6  12q13.13 | 343-754  692-1110  643-1064 | 756  673  641 | 96%  98%  99% | 0.0  0.0  0.0 | 99%  95%  94% |
| SR23 | pSRC39 | GU433085 | 356 | Spleen | 1) Bos taurus ubiquilin 4 (UBQLN4), mRNA >gb|BC126744.1| Bos taurus ubiquilin 4, mRNA  2) PREDICTED: Pan troglodytes similar to Ubiquilin 4 (LOC736474), mRNA  3) Homo sapiens ubiquilin 4 (UBQLN4), mRNA | NM_001080295.1  XR_020026.1  NM_020131.3 | mRNA =1446  mRNA =3588  mRNA =3572 | 3  3  1q21 | 1-348  68-414  69-415 | 632  536  536 | 97%  97%  97% | 3e-178  3e-149  3e-149 | 99%  94%  94% |
| SR26 | pSRC42 | GU433088 | 565 | Spleen | 1) Bos taurus ATP-binding cassette, sub-family D (ALD), member 1 (ABCD1), mRNA  2) PREDICTED: Equus caballus similar to ATP-binding cassette sub-family D member 1 (Adrenoleukodystrophy protein) (ALDP) (LOC100058868), mRNA  3) Homo sapiens ATP-binding cassette, sub-family D (ALD), member 1 | NM_001046190.1  XM_001491684.1  NM_000033.3 | mRNA =3563  mRNA =2199  mRNA =3697 | X  X  Xq28 | 1872-2417  1470-2016  1860-2406 | 948  756  728 | 96%  96%  96% | 0.0  0.0  0.0 | 97%  91%  90% |
| SR29 | pSRC45 | GU433091 | 674 | Testis | 1) PREDICTED: Bos taurus similar to Testis-specific Y-encoded protein 1 (Cancer/testis antigen 78) (CT78) (LOC786811), partial mRNA  2) Bos taurus Y Chr CH240-127C20 (Children's Hospital Oakland Research | XM_001254382.2  AC234853.4 | mRNA =1064  Gene length =130178 | Y  Y | 383-1041  4954-5212 | 1068  396 | 97%  88% | 0.0  1e-106 | 95%  98% |
| SR32 | pSRC48 | GU433094 | 327 | Testis | 1) PREDICTED: Bos taurus similar to polyhomeotic 1-like (PHC1), mRNA  2) PREDICTED: Canis familiaris similar to polyhomeotic 1-like, transcript variant 3 (LOC610167), mRNA  3) Homo sapiens polyhomeotic homolog 1B (Drosophila) (PHC1B) pseudogene on chromosome 12 | XM_590962.4  XM_861897.1  NG_009754.1 | mRNA =3819  mRNA =3294  Gene length =5458 | 5  27  12q13.2 | 898-1214  1044-1360  1202-1518 | 558  448  409 | 96%  96%  96% | 5e-156  1e-122  6e-111 | 98%  92%  90% |
| SR37 | pSRC54 | GU433100 | 342 | Testis | 1) Bos taurus latrophilin-1 type aa mRNA, complete cds  2) PREDICTED: Pan troglodytes similar to KIAA0821 protein (LOC455777), mRNA  3) Homo sapiens latrophilin 1 (LPHN1), transcript variant 1, mRNA | AF111097.1  XR_024518.1  NM_001008701.2 | mRNA =5161  mRNA =8331  mRNA =7874 | --  19  19p13.2 | 3681-3356  4007-3682  3548-3223 | 558  481  475 | 95%  95%  95% | 6e-156  1e-132  6e-131 | 97%  93%  92% |
